# Impact attribution of the March 2022 Antarctic heatwave reveals amplification by cloud feedbacks and increased future meltwater

**DOI:** 10.1038/s43247-026-03485-0

**Published:** 2026-04-16

**Authors:** Sergi González-Herrero, Pranab Deb, Sihan Li, Daniel Argüeso, Rainette Engbers, Michael Matějka, Nander Wever, Michael Lehning

**Affiliations:** 1 WSL Institute for the Snow and Avalanche Research SLF, Davos, Switzerland; 2 Centre for Ocean, River, Atmosphere and Land Sciences (CORAL), Indian Institute of Technology, Kharagpur, India; 3 School of Geography and Planning, University of Sheffield, Sheffield, UK; 4 Department of Physics, University of the Balearic Islands, Palma, Spain; 5 Environmental Engineering Institute, Ecole Polytechnique Fédérale de Lausanne, Sion, Switzerland; 6 Department of Geography, Masaryk University, Brno, Czech Republic; 7 Meteoswiss, Zurich, Switzerland

**Keywords:** Attribution, Cryospheric science

## Abstract

Antarctic heatwaves driven by atmospheric rivers are emerging as high-impact extremes, yet the role of climate change in amplifying such events remains uncertain. Here we investigate the climate change contribution to the March 2022 East Antarctic heatwave using pseudo-global warming experiments with the snow-atmospheric coupled model CRYOWRF. By comparing present-day and preindustrial storylines, we identify that under current climate, cloud and water-vapor radiation feedbacks non-linearly amplify near-surface warming by up to 10 °C (equivalent to 25% of the amplification) relative to preindustrial conditions. These feedbacks are likely underrepresented in global climate models due to their use of hydrostatic dynamics, and poor cloud representation over coarse resolutions. Future warming further intensifies this amplification, particularly along the coast, where firn air content is depleted, meltwater percolates, and ice lenses thicken. Such melting conditions threaten to accelerate surface mass loss and destabilize the fringing ice shelves. Our results reveal a key amplification pathway for Antarctic extremes, with potentially far-reaching implications for ice-sheet stability.

## Abstract (web summary)

Cloud and water-vapor radiation feedbacks amplified temperatures during the March 2022 Antarctic heatwave, which is likely underrepresented in global climate models, according to pseudo-global warming experiments with the snow-atmospheric coupled model.

## Introduction

In recent years, Antarctica has experienced a series of extreme events associated with atmospheric rivers (ARs) with significant impacts on the firn layer of the ice sheet^1^. In February 2020, the temperature at Esperanza station on the Antarctic Peninsula reached 18.3 °C, breaking the Antarctic continental temperature record. This event was driven by an AR and a subsequent foehn event on the leeside of the Antarctic Peninsula mountain range^2–4^, which enhanced the turbulent sensible flux and radiation over the ground^3^. In February 2022, another AR in the Antarctic Peninsula region set new local records, producing widespread surface melt across 52% of the region^5^. In March of the same year, yet another AR caused widespread warming across the East Antarctic ice sheet, setting new temperature records over an estimated 3.3 million km^2^ area and producing notable impacts on the SMB, ice sheet processes and polar ecosystems^6–9^.

This series of AR-driven heatwaves raised critical questions about whether they have been intensified by climate change, and if the projected further warming during this century will further exacerbate the impacts of such events on the Antarctic ice sheet. The Antarctic record-breaking event of 2020 was the first extreme event on the continent subjected to a formal attribution study^4^. It was assessed using an analog approach^10,11^, comparing a set of circulation-constrained analogs in the historical record. The same methodology was subsequently applied to the 2022 event in the Antarctic Peninsula^5^. Both studies quantified the contribution of climate change to be ~0.4 °C during the heatwave period, representing ~25% of the historical magnitude of comparable analog events. For the March 2022 event, a storyline approach was used. Instead of relying on event probabilities, the authors nudged a global climate model circulation toward observations under different forcing scenarios^6^ and examined their differences. This experiment suggested that the heatwave’s maximum temperatures were intensified by ~2 °C by anthropogenic amplification since the preindustrial period, equivalent to about 6 % of the total magnitude of the simulated 30 °C anomaly event.

These event-scale studies provide first insights into the amplification of Antarctic heatwaves by climate change, in contrast to the more gradual, long-term warming signal emerging over Antarctica^12^. However, several uncertainties remain due to potential limitations in the methodologies. For example, the analog method assesses only the changes in background temperature under similar synoptic configurations, without directly accounting for the physical drivers of the AR-induced warming. The relatively short observational period in Antarctica also limits the availability of suitable analogs for reproducing the atmospheric flow patterns of the event^13^. In the case of the storyline approach, adding excessive nudging can constrain the model variability, suppressing feedbacks and non-linear interactions that can naturally develop in free-running simulations^14–16^.

Importantly, the limited number of event attribution studies in Antarctica have so far focused on large-scale atmospheric drivers, without accounting for coupled surface-atmosphere feedbacks and non-linear interactions, such as cloud-radiation processes, that can lead to ‘exceptional’ amplifications as occurred in March 2022. To analyze how this record-shattering event was produced, we performed a series of storylines using pseudo-global warming (PGW) simulations^17,18^ with the coupled snow-atmosphere model CRYOWRF^19^. This model contains a detailed description of the firn layer and blowing snow processes, enabling it to capture key cryosphere–atmosphere interactions. These processes can strongly influence feedbacks such as changes in firn air content (FAC)^20,21^, slush formation^22^, ice slab growth^23^ and moisture and turbulent fluxes during blowing snow events^24^, which are critical for understanding extreme events in Antarctica. This allows us to construct physically consistent evolutions of the event, i.e. storylines, that account for possible atmosphere-cryosphere feedbacks during the March 2022 event and to assess their impacts on the firn layer. This is particularly relevant in the current context with surface meltwater increasing in East Antarctica^25,26^ and projected to become more widespread^27,28^, raising major concerns about hydrofracture and ice-shelf stability^29^.

Our storylines include a factual (current) simulation driven by the ERA5 reanalysis and three counterfactual simulations driven by historical ERA5 conditions modified by ‘delta’ changes corresponding to: (a) preindustrial conditions and (b) the late 21st century conditions under the SSP2-4.5 and SSP5-8.5 shared socioeconomic pathways (see Methods). A similar approach has been previously used to examine the current and future changes in tropical and polar cyclones^30,31^ among other meteorological events. Our approach allows us to attribute, for the first time, short-term firm responses to climate change.

## Results

### Storyline attribution highlights the role of the water vapor and cloud radiative feedback in amplifying the event

**Fig. 1:**
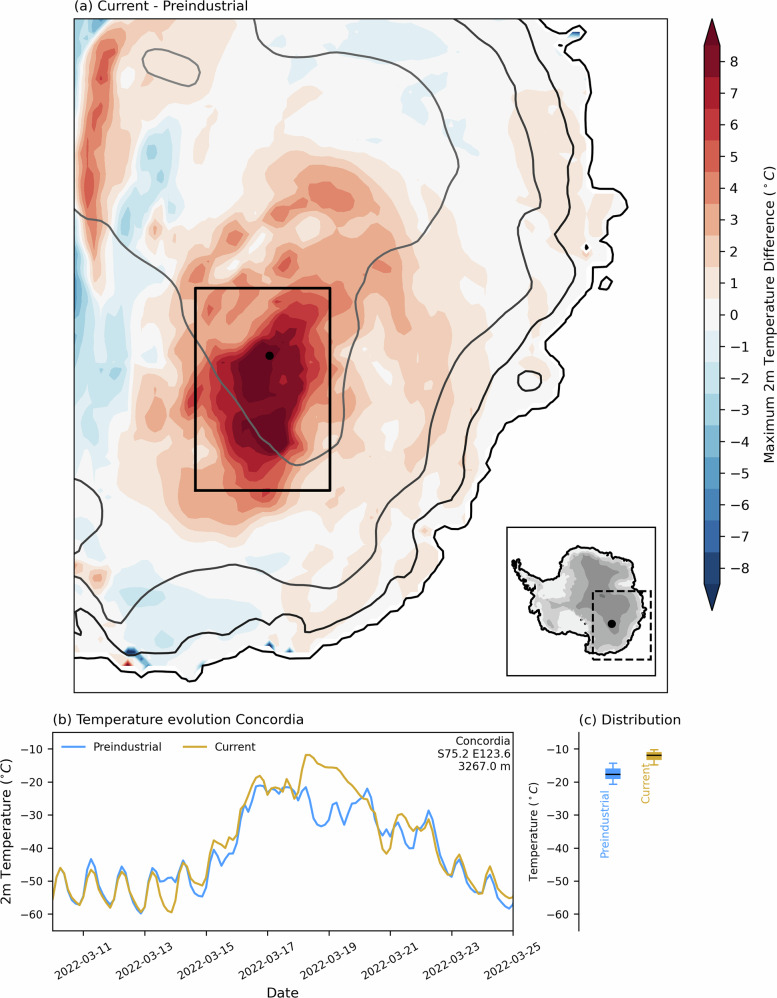
Temperature amplification by climate change during 17–19 March 2022. **a** Temperature amplification (in °C) between the factual (current) simulation and the preindustrial simulation. Black dot indicates the position of the Concordia station. The black rectangle depicts the Dome C region. The inset shows the location of the map inside Antarctica. **b** Temperature evolution at the nearest grid point to Concordia station (model coordinates S75.2 E123.6 at 3267 m height) for the different simulations. **c** Distribution of the maximum temperatures inside the Dome C region for the different simulations.

Our findings show that the March 2022 heatwave event was markedly amplified over Dome C under current climate relative to the preindustrial period (Fig. 1a), with a maximum amplification (difference between the maximum temperature in both experiments) averaging 5.4 ± 0.2 °C in a wide region centered near Concordia station (Fig. 1c) and exceeding 8 °C at the south of the Dome. This is equivalent to an amplification of 15–25% of the heatwave temperature anomaly (Supplementary Fig. 3.1). Temperature timeseries for the current and preindustrial experiments at Concordia (Fig. 1b) show a similar temperature increase during 15–16 March, with temperatures reaching values around −20 °C. While the current simulation maintains the temperature increase until 18 March, reaching a maximum temperature of −12 °C, the preindustrial simulation stabilizes from 17 March onward. It is between 18 and 20 March when the two temperature time series diverge in the two experiments, marking the period of amplification.

We examined the drivers that lead to the amplification of the temperature around Dome C by comparing the current and the preindustrial simulations. During the diverging phase of the event (18 March), the temperature tendency in the lower atmosphere (~20–80 m above the dome) was 0.22 ± 0.02 °C h^−^^1^ higher in the current simulation with respect to the preindustrial one. Analysis of individual contributors reveals enhanced contributions from horizontal temperature advection (0.68 ± 0.07 °C h^−^^1^) and diabatic heating (0.72 ± 0.17 °C h^−^^1^), which were partially offset by adiabatic cooling (−1.18 ± 0.19 °C h^−^^1^) (Table 1, Supplementary Fig. 1.2a, b). Low level moisture increased by 0.6 g kg^−^^1^ in the current simulation with respect to the preindustrial one, primarily driven by horizontal advection (0.09 ± 0.01 g kg^−^^1^ h^−^^1^), being partially offset by reduced vertical advection (−0.06 ± 0.02 g kg^−^^1^ h^−^^1^) from higher levels (Table 1, Supplementary Fig. 1.2c, d and Supplementary Fig. 1.3). Overall, these results suggest that temperature amplification over Dome C arises from approximately equal contributions of horizontal advection and diabatic effects, concurrent with a large horizontal moist advection.

**Table 1:** Changes for different heatwave variables between the preindustrial and current simulations in the Dome C region

		Variable	Change	Unit	*p* value
			(Current–preindustrial)		
Atmospheric variables	17–19 Mar 2022	Max temperature	5.39 ± 0.19	°C	<0.001
		PW	0.37 ± 0.02	kg m^−2^	<0.001
		LWP	0.84 ± 1.27	g m^−^^2^	<0.001
		IWP	0.69 ± 0.29	g m^−^^2^	<0.001
Energy balance components	17–19 Mar 2022	LWinc	15.95 ± 1.93	W m^−^^2^	<0.001
		LWout	14.95 ± 0.72	W m^−^^2^	<0.001
		SWinc	−2.92 ± 1.35	W m^−^^2^	<0.001
		SWout	−2.03 ± 1.10	W m^−^^2^	<0.001
		SH	1.40 ± 1.14	W m^−^^2^	0.02
		LH	0.52 ± 0.20	W m^−^^2^	<0.001
		Total	2.04 ± 0.16	W m^−^^2^	<0.001
Tendency components	18 Mar 22	HAdvT	0.68 ± 0.07	°C h^−^^1^	<0.001
		VAdvT	−1.18 ± 0.19	°C h^−^^1^	<0.001
		Diab	0.72 ± 0.17	°C h^−^^1^	<0.001
		TendT	0.22 ± 0.02	°C h^−^^1^	<0.001
		AdvQ	0.09 ± 0.01	g kg^−^^1^ h^−^^1^	<0.001
		VAdvQ	−0.06 ± 0.02	g kg^−^^1^ h^−^^1^	<0.001
		Source/Sink	0.00 ± 0.02	g kg^−^^1^ h^−^^1^	0.92
		TendQ	0.03 ± 0.00	g kg^−^^1^ h^−^^1^	<0.001

**Fig. 2:**
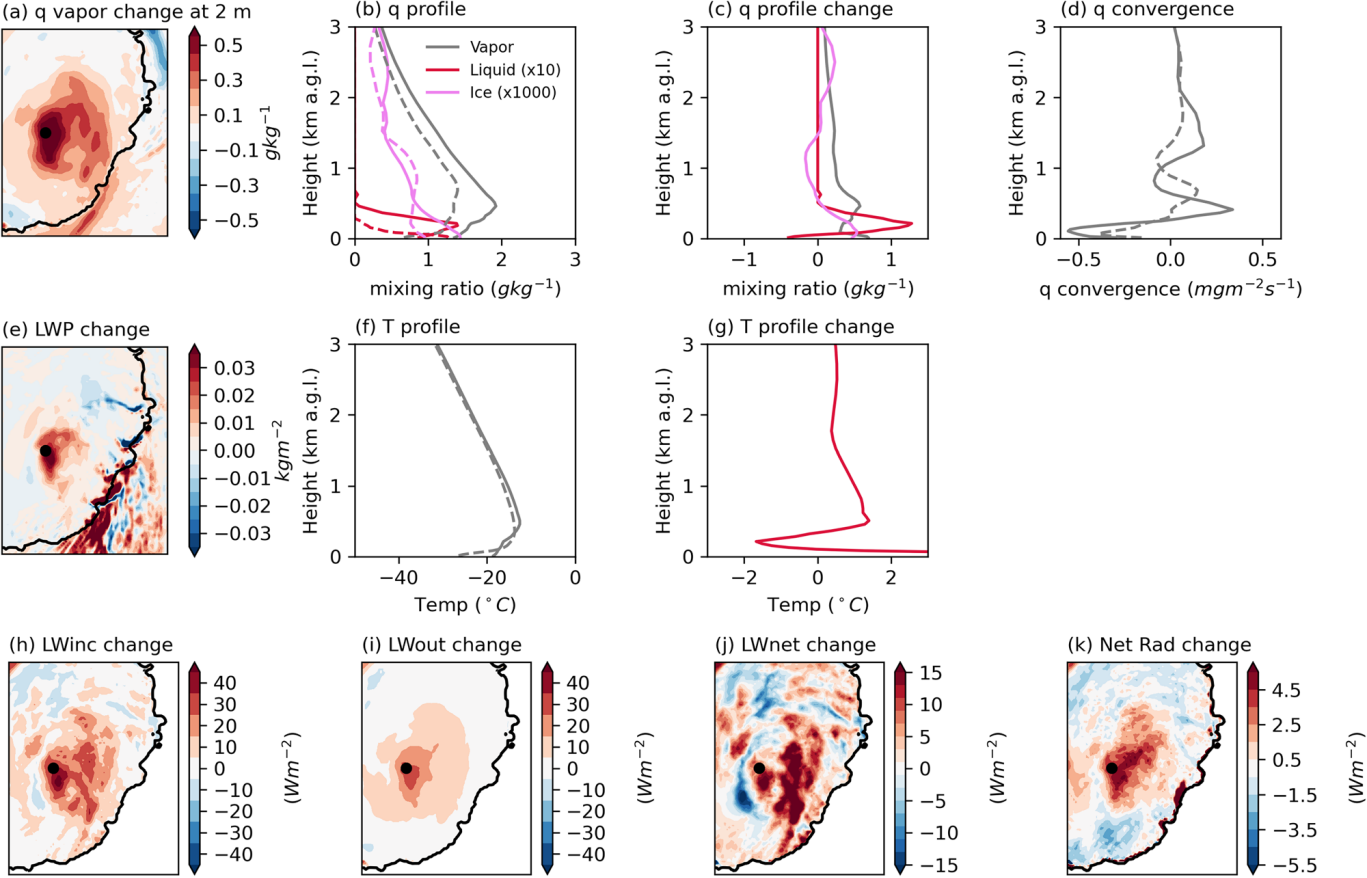
Physical processes that contributed to the temperature amplification during 17–19 March 2022. Changes in the 17–19 March 2022 averaged values between the current and the preindustrial simulations of **a** vapor mixing ratio (q vapor in g kg^−^^1^), **e** liquid water path (LWP in kg m^−^^2^), **h** long wave incoming radiation (LWinc in Wm^−^^2^), **i** long wave outcoming radiation (LWinc in Wm^−^^2^), **j** long wave net radiation (LWnet in Wm^−^^2^), **k** net surface energy (Net Rad in Wm^−^^2^), **c** vertical profiles of q vapor (grey in g kg^−^^1^) and q liquid (red in 10 g kg^−^^1^) and q ice (magenta in 1000 g kg^−^^1^) and **g** vertical profile of temperature (red in °C) at the nearest point to Concordia station. **b**, **f** as **c** and **g** but showing the values for the current (solid lines) and preindustrial (dashed lines) simulations. **d** Vertical profile of moisture convergence for the current (solid lines) and preindustrial (dashed lines) simulations. **a**, **d** red (blue) colors represent an increase (decrease) of mixing ratio and LWP during the preindustrial simulation, while in **g**–**j**, red (blue) colors represent an increase (decrease) of incoming radiation towards the surface. The black dot in the maps represents the position of the Concordia station. Vertical profiles are a function of height above ground level (m a.g.l.).

We further explored the drivers of diabatic warming by analyzing the changes in radiative components associated with changes in the vertical water component profiles. Figure 2a shows that the spatial pattern of the water-vapor and cloud mixing ratios at 50 m above the surface averaged over the heatwave period (17th–19th of March; see Supplementary Fig. 2.1) closely resembles the amplified temperature changes in Fig. 1a. Water vapor mixing ratio profiles at Concordia (Fig. 2b, c) reveal a 0.5 g kg^−^^1^ increase in low-level water vapor ratio in the current simulation compared to the preindustrial simulation, despite the increased low-level divergence (Fig. 2d). The current simulation also produces a thick (0–500 m) layer with supercooled liquid droplets (q liquid profiles in Fig. 2b, e) over Concordia. This layer is also present in the preindustrial simulation but is thinner (0–200 m) than in the current simulation with the droplets closer to the surface and may be associated with local production due to enhanced moisture convergence aloft (Fig. 2d). The increase in droplets is concentrated over the Dome C as revealed by the spatial map of the vertically integrated cloud liquid water (LWC in Fig. 2d).

Changes in both water vapor and cloud liquid water influences the temperature profile (Fig. 2f, g) by warming the surface and the lower atmospheric layers through enhanced downward longwave radiation emission from cloud and water vapor (Fig. 2h). The resulting surface warming increases the outgoing longwave radiation (Fig. 2i), but this is not sufficient to offset the incoming longwave radiation (Fig. 2j) producing a positive net energy change on the surface of 2.0 ± 0.2 Wm^−^^2^ (Table 1; Fig. 2i). Higher cloud water content slightly reduces both the incoming and outgoing net short-wave radiation at the surface by −2.9 ± 1.4 and 2.0 ± 1.1 Wm^−^^2^, respectively (Table 1, Supplementary Fig. 1.4). But the major changes are seen in longwave incoming radiation which increases by 16.0 ± 1.9 Wm^−^^2^. Although ARs are known to enhance heat fluxes^32^, the global warming response leads to a redistribution of this flux, with reduced sensible heat uptake on the windward side and enhanced release on the leeward side (Supplementary Fig. 1.4d). The result is an average positive effect of 1.4 ± 1.1 Wm^−^^2^ over the Dome C region (Supplementary Fig. 1.4p). In contrast, changes in surface latent heat flux are small, contributing only 0.5 ± 0.2 Wm^−^^2^ over the dome (Supplementary Fig. 1.4q). Thus, the effect of water-vapor and cloud liquid water changes is expressed primarily through changes in longwave radiation, leading to surface warming that is largely compensated by increased outgoing longwave radiation of 15.0 ± 0.7 Wm^−^^2^. Nevertheless, a net positive surface energy anomaly persists over Dome C (Fig. 2i). The increase in liquid and vapor water is in turn driven by enhanced horizontal moisture advection at low levels (Supplementary Fig. 1.2c, d), combined with rising temperatures.

**Fig. 3:**
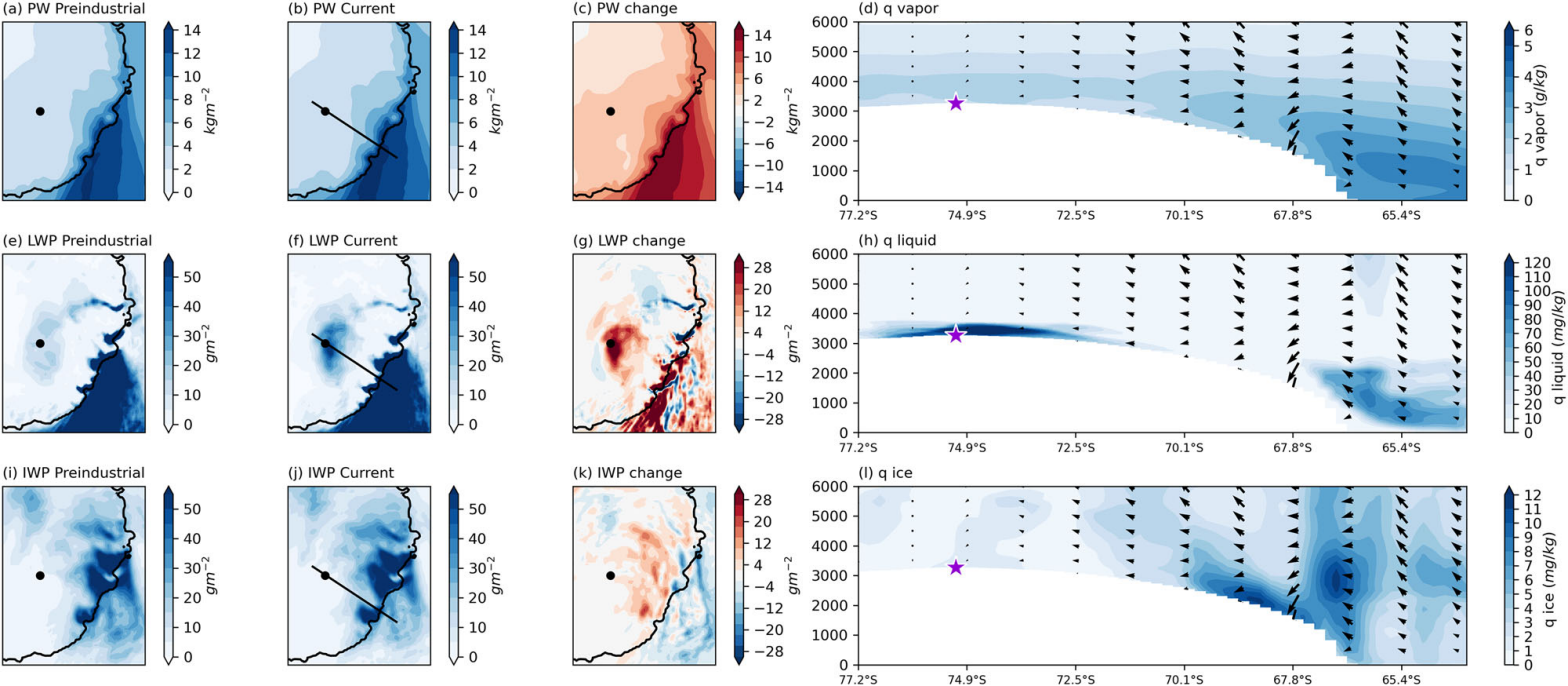
Changes in vertically integrated water components between the current and preindustrial simulations and corresponding cross sections of mixing rations for the current simulation. **a**–**c** Precipitable water vapor (PW; kg m^−^^2^), **e**–**g** liquid water path (LWP; g m^−^^2^), and **i**–**k** ice water path (IWP; g m^−^^2^), for the **a**, **e**, **i** preindustrial simulation, **b**, **f**, **j** current simulation, and **c**, **g**, **k** change between both simulations. **d**, **h**, **l** Vertical profiles of vapor, liquid and ice mixing ratio along the 123 °E meridian. Values are averaged between 17 and 19 March 2022.

A relevant question is why liquid droplets are present over the dome but not along the ascent path (Fig. 3f, h), where only ice-phase hydrometeors are found (Fig. 3j, l). Cloud particles associated with the AR are initially formed primarily as liquid over lower latitudes and gradually transition to mixed-phase near the Antarctic coast (Fig. 3f, h, j, l). As the air penetrates inland and cools, the liquid phase is largely depleted and converted into ice through freezing and deposition processes. During the ascent over the Antarctic Plateau, the continued large-scale lifting further cools the air mass, progressively reducing its moisture-holding capacity (Fig. 3b, d). Over the dome, weak winds and moisture convergence promote supersaturation with respect to both liquid water and ice, favoring the in-situ formation of supercooled liquid droplets from advected water vapor in a low ice nucleation particle environment. In the simulations, this behavior is influenced by the temperature-dependent ice nucleation parameterizations of the Morrison microphysics scheme. Although this may lead to quantitative uncertainties in phase partitioning, the resulting dominance of supercooled liquid water is physically plausible and consistent with observations over the Antarctic Plateau^33^. Observational studies indicate that extremely low ice nucleation particle concentrations suppress efficient heterogeneous ice nucleation^33,34^, allowing liquid droplets to persist and rely primarily on homogeneous freezing at very low temperatures^34,35^. Continuous vapor supply maintains these droplets, while ice growth and precipitation remain inefficient, producing a localized maximum in liquid water path over Dome C. Our simulations suggest that current warmer conditions enhance this effect with respect to the past (Fig. 3c, g, k).

In our simulations, the combined effect of these changes leads to a substantial increase of downward longwave radiation of 10–40 W m^−^^2^ over Dome C, with much weaker increases away from Dome C. While the exact partition between water vapor, ice clouds, and liquid clouds cannot be quantified without dedicated radiative perturbation experiments, the spatial coincidence of the longwave radiation maximum with enhanced liquid water path over Dome C (Fig. 3f) indicates that supercooled liquid clouds act as a key local amplifier of the near-surface warming. This interpretation is consistent with Arctic observations and modelling studies showing that increases in liquid water path of 13–43 g m^−^^2^, can lead to an increase of downward longwave radiation by 40 W m^−^^2^ over the surface^36^. Recent observational studies at Dome C further demonstrate that cloud liquid water content increases logarithmically with temperature, producing a similar radiative impact in downward longwave radiation^37,38^. These findings provide strong support for the hypothesis that supercooled liquid droplets may be the main driver in the non-linear temperature amplification.

In summary, our simulations show that water–vapor and cloud liquid water increase is directly responsible for the climate change-driven temperature amplification during this event beyond the linear change given by CMIP6 models (Supplementary Fig. 4.1).

### Impact of Antarctic heatwave amplification on the firn layer over Dome C

The exceptional heatwave amplification raises concerns about its potential impact on surface snow and the firn layer, and the implications for Antarctic mass balance. Previous research has shown that AR-associated heatwaves have a considerable impact on the snow layer and emphasized the need for improved land-surface model to properly simulate these effects^39^. Using CRYOWRF with an advanced snow/firn land-surface model, we assess for the first time the impacts of Antarctic heatwave amplification on the cryosphere. Our simulations reveal an increase in the surface mass balance (SMB) by more than 10 mm w.e. on the windward side of the AR [Supplementary Fig. 1.5n], primarily due to an increase in precipitation [Supplementary Fig. 1.5h]. This response is slightly modulated by the surface ridges and troughs of the coastal ice sheet^40^. While precipitation accounts for more than 90% of the SMB change in most of the domain, some coastal regions exhibit local changes in erosion, deposition and sublimation changes [Supplementary Fig. 1.5i–k], likely resulting in minor differences in snow redistribution between the two experiments. Along the Wilkes Coast, melt and refreeze processes dominate the SMB change, increasing by more than 25% compared to the preindustrial simulation [Supplementary Fig. 1.5l, m].

The increased energy input at the surface warms the snow in both simulations, but under the current climate, the amplified heatwave drives stronger warming that penetrates the firn down to 1 m below the surface in the days following the event at Concordia [Supplementary Fig. 1.6a]. Snow density timeseries shows a decrease in both simulations due to snowfall on 16th March. After subsequent snow compaction, snow density increases above the initial conditions in the current simulation but remains lower in the preindustrial simulation [Supplementary Fig. 1.6b]. The lower density in the preindustrial case arises from the absence of the modest precipitation increase seen in the current climate, which limits snow loading and therefore reduces post-depositional compaction. While resulting density differences are relatively small, they modify the snow thermal gradient and firn physical properties, influencing firn metamorphism and the exchange of water isotopes with the atmosphere^41^.

**Fig. 4:**
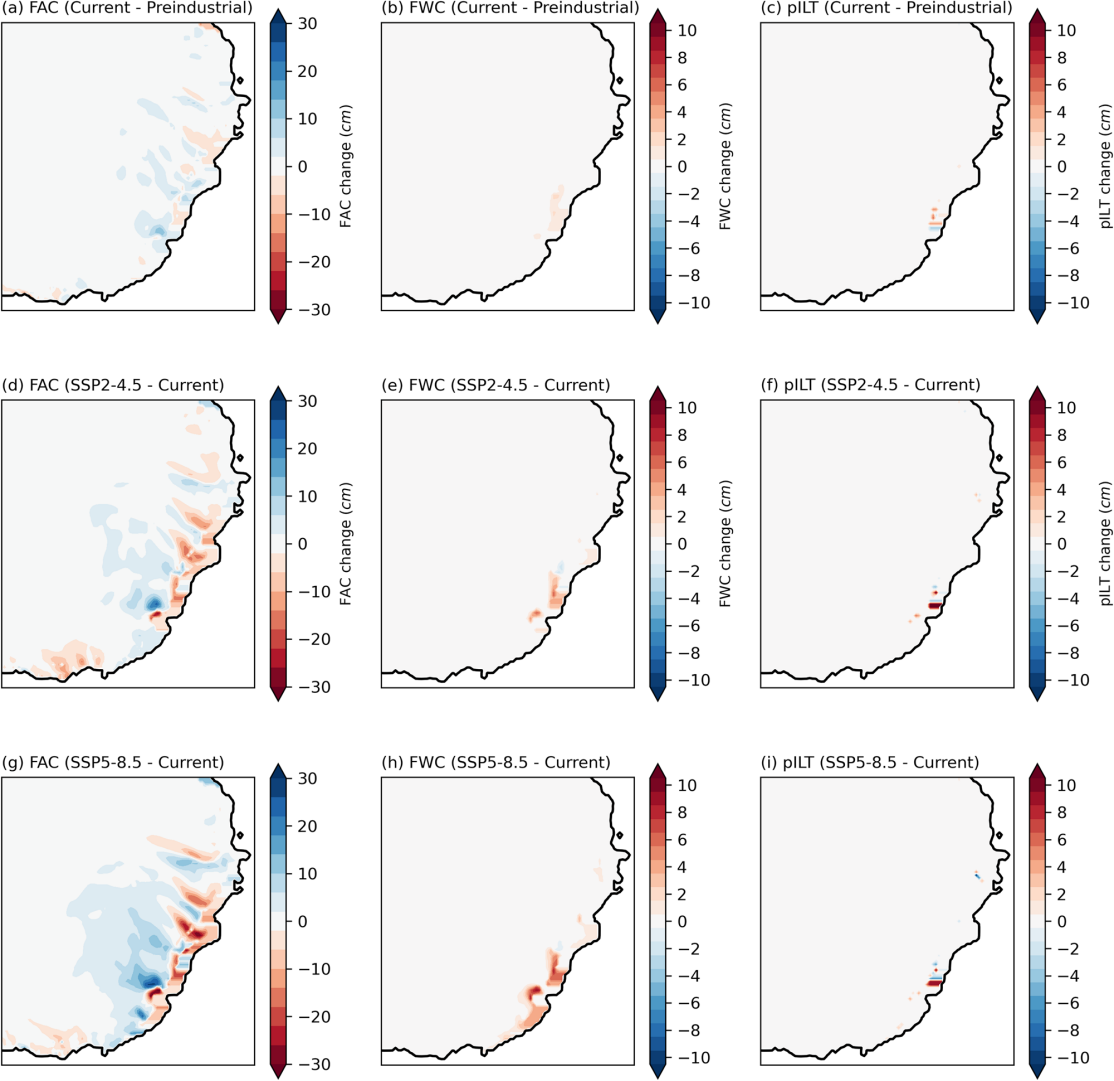
Firn impact scenarios for the event on 19 March by the end of the century. **a**, **d**, **g** Firn air content change. **b**, **e**, **h** Firn liquid water content change. **e**, **f**, **i** Potential ice lens thickness change. Changes show the difference on 19 March between the preindustrial and current simulations (**a**–**c**) and for the current and the future simulations under the SSP2-4.5 scenario (**d**–**f**) and SSP5-8.5 scenario (**g**–**i**) by the end of the century.

These structural changes in the firn layer are accompanied by a broader firn response, including changes in FAC, which provides an integrated measure of pore space and controls the capacity of the firn to store meltwater and buffer surface meltwater inputs^20,42,43^. Consistent with previous studies of this event using the SNOWPACK model^8^, our model simulates a strong increase in FAC during the event at Wilkes Land, exceeding 30 cm in coastal regions [Supplementary Fig. 1.7a]. Near the coast, rainfall combined with high temperatures introduces liquid water into the firn [Supplementary Fig. 1.7b], and refreezing of this water led to the formation of ice lenses [Supplementary Fig. 1.7c], which can hinder water percolation^21^. While the event itself drives substantial changes in FAC, our attribution analysis indicates that climate change has only a minor effect on firn air [Fig. 4a], but still produces a two-fold increase in firn liquid water near Wilkes Land from 0.008 Gt in the preindustrial conditions to 0.014 Gt in the current conditions [Table 2; Fig. 4c]. This small addition of liquid water is sufficient to slightly increase the potential ice lens thickness (pILT) under current climate conditions relative to preindustrial conditions [Fig. 4f].

**Table 2:** Maximum FWC during the event and pILT in Wilkes Land at the end of the event for different simulations

	Maximum FWC	Ice thickness
Preindustrial	0.008 Gt	1.159 km^3^
Current	0.014 Gt	1.161 km^3^
SSP2-4.5	0.03 Gt	1.172 km^3^
SSP5-8.5	0.079 Gt	1.169 km^3^

### Future scenarios shift the amplification towards the coast with further intensified impacts on the firn

**Fig. 5:**
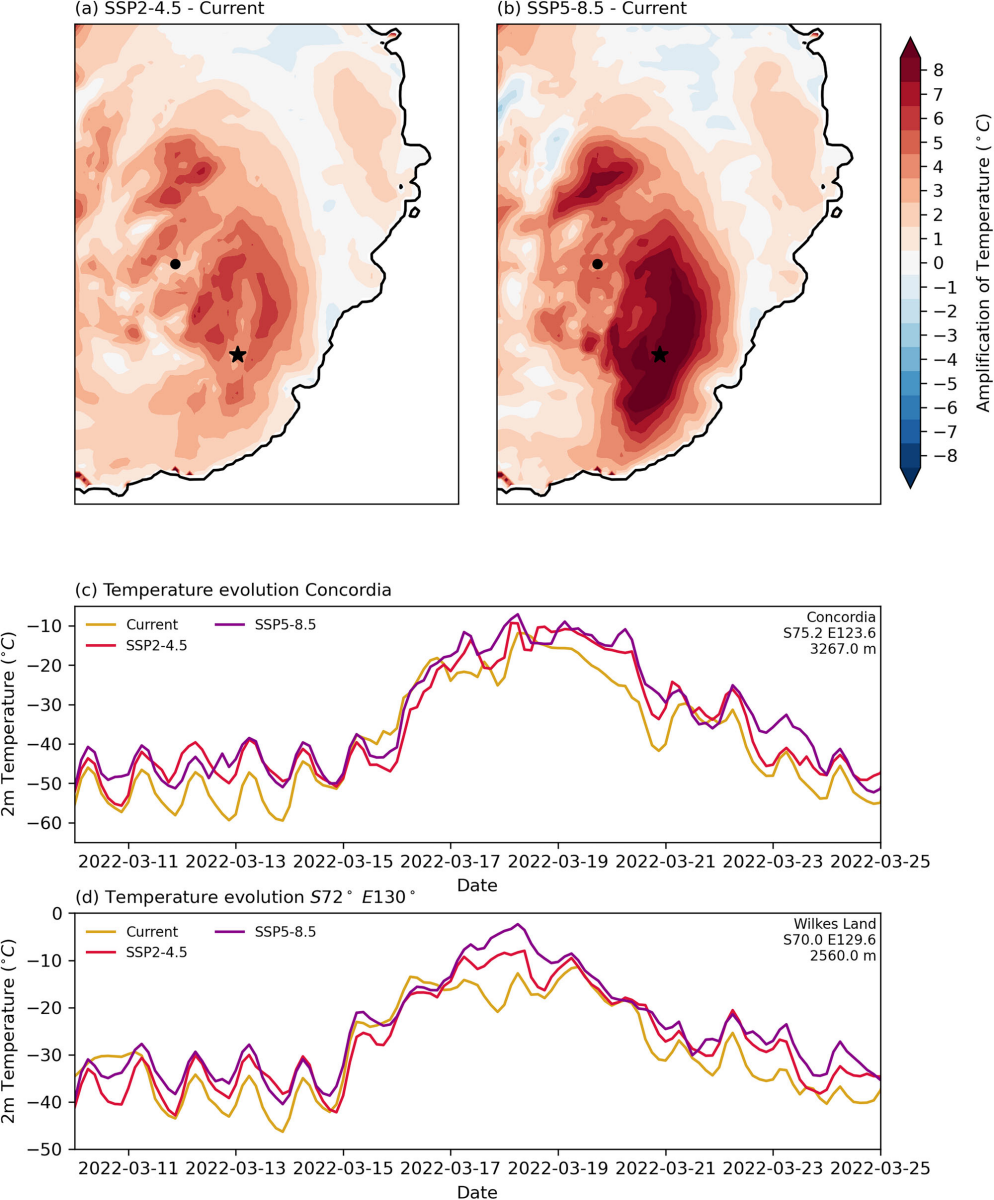
Future scenarios for the event during 17–20 March 2022 by the end of the century. **a**, **b** As Fig. 1a but for the changes between the current and the future simulations for the SSP2-4.5 scenario (**a**) and SSP5-8.5 scenario (**b**) by the end of the century. **c**, **d** Temperature evolution at the nearest grid point to Concordia station (model coordinates S75.2 E123.6 at 3267 m height; dot in **a**, **b**) for the different future scenarios, and at a point in Wilkes Land (model coordinates S70.0 E129.6 at 2560 m height; star in **a**, **b**).

Thermodynamically driven temperature amplification during the AR by the end of the century in two different scenarios indicate a further intensification under future warming, with regional shifts (Fig. 5; Supplementary Fig. 3.2). Notably, while current amplification is centered over the interior domes, future simulations by the end of the century in SSP2-4.5 and SSP5-8.5 scenarios, show limited warming over these domes with respect to the present. Instead, they show a pronounced amplification extending toward the coast, particularly under high-emission scenarios. In these simulations, changes are not only driven by the increase of supercooled liquid water but by increases in all the vertically integrated water components, including water vapor and ice cloud droplets (Supplementary Figs. 1.8 and  1.9), due to a stronger horizontal moisture advection towards the continent and higher extension of ice clouds (Supplementary Fig. 1.10) probably driven by a warmer atmosphere and reduced sea ice extent. Importantly, the spatial pattern of non-linear temperature amplification evolves with the background climate state. In the transition from preindustrial to current conditions, amplification is strongest over the cold interior, including Dome C. In contrast, in future simulations relative to the current climate, the non-linear amplification progressively weakens over the plateau and shifts toward lower-elevation regions, first between the interior and the coast (SSP2-4.5) and ultimately becoming confined to coastal areas under stronger warming (SSP5-8.5). The return to the linear background warming signal suggests a saturation of cloud-radiative feedbacks, and the progression towards the coast suggests that cloud–radiative feedbacks operate most efficiently within a specific temperature and moisture regime, which migrates spatially as the Antarctic climate warms (Fig. 5a, b).

As the heatwave amplification shifts toward the coast, its impacts also do the same. FAC decreases substantially along the coast after the event, with reductions up to 30 cm in the SSP5-8.5 scenario, while modest increases are still observed in the interior of Wilkes Land (Fig. 4d, g). Large reductions next to the coast are driven by rainfall and meltwater percolation, which increase settling rates and reduce pore space in the firn. Similarly, firn liquid water content (FWC) rises by 4 cm in the SSP2-4.5 scenario and by up to 10 cm in the SSP5-8.5 scenario, with the affected areas expanding accordingly in warmer scenarios (Fig. 4e, h). The associated meltwater production increases dramatically and non-linearly. While the current simulation produces only 0.012 Gt, future projections show increases by factors of 2.4 and 5.6 under SSP2-4.5 and SSP5-8.5, respectively (Table 2). This single cold-season event alone would generate up to 0.079 Gt of meltwater by the end of the century under the SSP5-8.5 scenario. The excess liquid water further enhances pILT within the firn, increasing ice-lens volume by up to 0.01 km³ (Table 2; Fig. 6f, i). These values represent lower bounds, as a fraction of the meltwater has not yet fully percolated by the end of the simulation and would continue to refreeze with additional settling time. The resulting reduction in firn permeability favors meltwater ponding and promotes hydrofracturing. These processes are known to weaken ice-shelf stability^20^, highlighting the potential consequences that future AR-driven heatwaves may exert along Antarctic coasts.

## Discussion

The role of low-level liquid and mixed-phase clouds as a key feedback mechanism on the polar surface radiation balance is well recognized^36,44–46^. However, considerable uncertainty remains regarding their contribution in Antarctica, where cloud microphysics are still poorly understood^47^. Compared to the Arctic, liquid-bearing clouds are less prevalent and persistent in Antarctica due to colder temperatures and lower nucleating particle concentration^48^, appearing more frequently in West Antarctica during the austral summer^49^, where they can enhance surface melting near the coast^50^. Rising temperatures, however, may trigger amplification processes currently common in the Arctic but largely absent in Antarctica^51^. The case-study presented here, using a 27-km-resolution non-hydrostatic model CRYOWRF, suggests that this mechanism is already affecting the Antarctic plateau, producing non-linear temperature increases in regions where clouds are enhanced. Indeed, our PGW simulations show a much stronger warming effect than reported in the NCAR CESM1-CAM5^6^ storylines of the same event, yielding markedly improved representation of the event magnitude compared with NCAR CESM1-CAM5 storylines (model evaluation of Methods section). In CRYOWRF, the peak temperature in the current climate simulation reaches −11.9 °C, close to the −11.6 °C in the observations [Supplementary Fig. 1.1], whereas NCAR CESM1-CAM5 simulations reported a peak of −20 °C, much lower than the observed. This suggests that the model complexity plays an important role in quantifying the amplification.

In typical Global Climate Models (GCMs) like NCAR CESM1-CAM5, coarse grid resolution and the hydrostatic approximation strongly simplify the representation of vertical motions, requiring cloud microphysics and turbulent processes to be parameterized over large grid volumes. In our simulations, vertical moisture advection increase is of comparable magnitude to horizontal advection during the event (Table 1), enabling enhanced formation and persistence of supercooled liquid water clouds within shallow inversion layers—processes that would be strongly damped in a hydrostatic model. These processes, which are among the most poorly constrained aspects of the models, lead to suppression of key processes that high-resolution non-hydrostatic models can explicitly resolve^52^. In addition, the lower vertical resolution may suppress key layers and atmospheric inversions. These limitations are especially relevant in very strong advective events as seen in ARs, which transport large amounts of moisture into Antarctica and promote cloud formation within strong low-level inversions^53^, favoring the persistence of supercooled liquid water clouds that enhance longwave downward radiation^54^. The difference between the simulations presented here and the NCAR CESM1-CAM5^6^ simulations suggests the need for accurate water-vapor and cloud representation in climate models to robustly estimate impacts of extreme events over Antarctica. Despite decades of progress in climate model development, accurately simulating clouds and representing mixed-phase clouds remains challenging^55^. As a result, cloud feedbacks are still a key source of uncertainty in climate sensitivity estimates in CMIP6 models^56,57^. For example, a recent study^58^ compared supercooled liquid-containing clouds in CMIP6 models with satellite observations and found that CMIP6 models consistently overestimate their occurrence, leading to light snowfall being produced too frequently. This excessive snowfall maintains an artificially bright snow surface over ice sheets enhancing albedo and causing GCM to underestimate surface melt. Furthermore, liquid water cloud presence does not necessarily translate into a realistic radiative response during extreme events. The non-linear surface warming identified here arises from the tight coupling between moisture advection, sharp low-level inversions, and the vertical confinement of supercooled liquid water clouds, which strongly enhances downward longwave radiation over short time scales. In contrast, parameterized cloud schemes in GCMs tend to distribute liquid water more diffusely in the vertical direction and produce more persistent cloud fields, weakening their interaction with near-surface inversions and smoothing the radiative response^59,60^. As a result, even when supercooled liquid clouds are present in nudged simulations with GCMs, their event-scale radiative amplification may be underestimated. These biases arise from poor representation of cloud microphysics, further highlighting the need for model improvements in cloud microphysics and sub-grid processes.

Our future storylines highlight that warming-driven increases in moisture advection by ARs, as projected in the end-of-the-century climate simulations^61^, can substantially enhance water vapor and cloud liquid and ice water paths. This, in turn, triggers non-linear temperature amplification and intensifies AR-associated heatwaves near the coast, consistent, as well, with broader evidence that surface melt can further enhance warming during extreme events^62^. While the current amplification produced only minor impacts on the firn near the coast, future amplifications are projected to promote firn air depletion, due to rainfall, melt, enhanced settling, liquid water percolation and refreezing, and ice lens formation^21,63^. The magnitude of these impacts is expected to increase under high-emission scenarios, highlighting the potential consequences of coastal heatwave amplification for the Antarctic cryosphere.

While our simulations highlight previously unidentified non-linear amplification mechanisms, we acknowledge that these results are based on a single set of high-resolution storyline experiments rather than an ensemble sample^64^. This inevitably limits a robust statistical assessment of the significance of the changes. Building large ensembles of cryosphere-atmosphere coupled models at such resolution over Antarctica remains computationally costly, but our approach provides physically consistent storylines that complement large-scale GCM ensembles. We therefore present these results as a process-based exploration of plausible responses. They should be interpreted as complementary to large-scale GCM ensembles, rather than probabilistic estimates bridging weather-scale processes with climate-scale projections.

Despite these limitations, our findings point to an important amplification mechanism largely absent from current GCMs. The resulting warming strongly affects the SMB and alters the temperature and density of snow and firn in the upper meter of the ice sheet. While impacts over the high interior domes remain limited to the SMB and the near-surface firn properties with persistently sub-zero temperatures, they become pronounced in coastal regions where temperatures approach the melting point^65^, allowing liquid water percolation to the firn. There, the identified amplification mechanism can drive sudden and non-linear temperature increases during AR-driven heatwaves, rapidly pushing the surface toward melt thresholds. In the case of the ice shelfs, an increased surface melting due to temperature amplification may lead to an increase of percolation and hydrofracturing and eventually drive ice shelf collapses^29,66^. The March 2022 event, linked to the collapse of the Conger Ice Shelf^8^, illustrates how such extremes can directly affect ice-sheet stability. While recent studies show that surface meltwater is already increasing in coastal East Antarctica^25,26^, our results indicate that future warming will further amplify heatwave extremes, generating additional snowfall but also meltwater and slush that can destabilize the fringing ice shelves beyond the current projections from GCMs. This enhanced vulnerability to extreme heatwaves in the future could trigger rapid, potentially irreversible changes in East Antarctica with far-reaching consequences^67^.

## Online methods

### Model set up

Four numerical experiments representing the extreme event on 15–20 March 2022 under different climate conditions were conducted with CRYOWRF^19^, to simulate the atmospheric-cryosphere interactions of the event. CRYOWRF combines the widely used non-hydrostatic model WRF^54^ with the snow-firn model SNOWPACK as the land-surface model^68^, including a state-of-the-art blowing snow module. CRYOWRF allows, therefore, detailed investigations into the changes of snow under different atmospheric conditions by theoretically representing the snow with an infinite number of layers simulated online with the atmospheric conditions and their two-way interactions. This model has been extensively evaluated in Antarctica, showing good agreement with SMB observations from 9660 locations, 148 atmospheric weather stations and two time series of drifting snow measurements in the continent^69^. The use of SNOWPACK as a land surface model is also to quantify firn variables, such as air depletion and meltwater volume^70^.

The model is set up as one domain covering all Antarctica at 27 km resolution. The simulation had 64 vertical levels up to 100 hPa, with 8 additional layers near the surface to resolve drifting snow saltation and up to 500 potential snow layers. A timestep of 90 seconds was used. Experiment set-up was performed with the configuration previously used and tested by Gerber et al.^69^ consisting of, in short, a planetary boundary layer (PBL) parametrized by the Mellor-Yamada-Nakanishi-Niino 2.5 level turbulent kinetic energy scheme with the scalar mixing option, a subgrid-scale turbulence parametrized using the Smagorinsky first order closure, and a precipitation and cloud microphysics parametrized using the 2-moment Morrison scheme adapted to polar atmospheric conditions. Atmospheric nudging is applied only for wind in the top 20 atmospheric layers. In the land surface model, SNOWPACK runs every 15 minutes and uses the Holstag stability correction for surface fluxes^71^, albedo parametrization by Munneke et al.^72^ and a water transport described by a bucket model. Blowing snow is represented by a classic double-moment hydrometeor model, which includes advection, turbulent diffusion of particles, sublimation and deposition.

The four experiments (as detailed below) were run over the 5-25 March 2022 period, with the first 10 days as a spin-up period to adjust the atmospheric conditions and the surface snow layers. The snow and firn initial profile included an additional 12-year spin-up with ERA5 as an atmospheric forcing data to align the snow-firn cover to the model physics processes. The physics and dynamics configurations of the model followed Gerber et al.^69^.

### Model evaluation

The ‘current’ run has been evaluated against surface temperature, as well as temperature and moisture profiles recorded by the Agenzia nazionale per le nuove tecnologie, l’energia e lo sviluppo economico sostenibile (ENEA) at Concordia station^73,74^, where the maximum temperature amplification was observed. The extensive measurements available at this site provide a detailed assessment of the model’s performance. Our model exhibits a warm bias during the stable initial period (10–15 March) and simulates the near-surface temperature inversion at a higher elevation than observed. Such discrepancies are common in models over the Antarctic plateau and primarily reflect limitations in representing extremely shallow inversions. However, both the duration of the heatwave and the maximum temperature in the current simulation align well with observations [Supplementary Fig. 1.1c]. This bias remains consistent throughout most of the temperature profile, indicating internal consistency in the model [Supplementary Fig. 1.1a] and is present after the heatwave, suggesting that it does not control the event amplification analyzed here. A comparison of the water vapor mixing ratio before and during the heatwave event shows that the model accurately simulates the water vapor advection driven by the AR [Supplementary Fig. 1.1b]. The only notable discrepancy that may affect our results arises from the height of the near-surface temperature inversion, which is simulated as higher than observed. This elevated inversion may influence the detailed coupling between clouds, moisture and the surface. However, the direction of this effect is not straightforward. While weaker surface inversion can enhance turbulent exchange and increase near-surface humidity and cloud formation near the ground, the same enhanced mixing can also spread the cloud layer over a deeper vertical extent, thereby reducing its radiative efficiency at the surface. Nevertheless, the model successfully captures the overall event dynamics and the primary drivers of the AR-induced warming, providing us with confidence in the results presented here.

### PGW experiments

The four experiments comprised a current control simulation and three PGW experiments with varying initial and lateral boundary conditions. The PGW counterfactual experiments represent a preindustrial simulation to calculate the climate change contribution and two future climate change scenarios to calculate a potential future event at the end of the 21st century. For the PGW experiments, initial conditions obtained from ERA5^75^ were adjusted using data from six CMIP6 models, calculating the delta differences using the PGWERA5WRF package^76^, after adapting it to polar regions and CRYOWRF. This includes the modified variables listed in Supplementary Table 4.1 with the notable inclusion of sea surface temperature and sea ice concentration. To avoid inconsistent physical feedback between the surface and the atmosphere, the initial snow temperature profile was modified by the delta change of the surface temperature in the CMIP6 models. All the other snow parameters remained unchanged, being modified only during the atmospheric spin-up process. The control simulation (i.e. current simulation) used the last 30-year period of the historical simulation (1985–2014) as a baseline. The preindustrial simulation used the first 30-year period (1850–1879) similarly to Parker et al.^23^. Future simulations represented the AR event at the end of the 21st century (2070–2099) under the SSP2-4.5 and SSP5-8.5 climate scenarios. The changes attributed to climate change are calculated by subtracting the preindustrial experiment from the control simulation. We define the event temperature amplification as:

1 $${\left.{T}_{{amplification}}\right|}_{{current}-{preindustrial}}({\scriptstyle{{\circ }}\atop}\!C)=max \left({T}_{i,{current}}\right)-max \left({T}_{i,{preindustrial}}\right)$$

and in percentage as:

2 $${\left.{T}_{{amplification}}\right|}_{{current}-{preindustrial}}=\frac{max \left({T}_{i,{current}}\right)-max \left({T}_{i,{preindustrial}}\right)}{max \left({T}_{i,{preindustrial}}\right)-{T}_{o,{preindustrial}}}\times 100$$

where *T*_*i*_ is the temperature in °C at every time step during the event with outputs every three hours and *T*_*0*_ is the temperature at the beginning of the event defined at 00 UTC on 15 March 2022. Because the PGW approach relies on a small number of deterministic simulations, it does not permit formal uncertainty quantification based on ensemble statistics. Instead, we assess the robustness of the simulated response by analyzing the distribution of the climate change response over the Dome C region (Fig. 1). This provides a quantitative measure to assess whether simulated changes exceed the internal spatial variability (see Table 1). Supplementary Table 4.2 and Supplementary Fig. 4.1 show the mean climate deltas over the whole model domain.

### Surface mass and energy balance

The surface mass and energy balance changes were calculated by subtracting the preindustrial from the current scenarios during the AR event defined between 17 and 20 March 2022. The energy balance analysis includes the PGW changes of the atmospheric components described by the equation:

2 $${TotalEnergy}={{LW}}_{{out}}+{{LW}}_{{in}}+{{SW}}_{{out}}+{{SW}}_{{in}}+{SH}+{LH}+P$$

where LW is the long-wave radiation, SW is the short-wave radiation, SH is the sensible heat flux, LH is the latent heat flux, and P is the energy from precipitation in Wm^−^^2^. The sign convention used is such that the surface energy fluxes are positive when directed towards the surface. From these components, the radiative fluxes are calculated by WRF and used as boundary conditions (longwave) or volume source term (shortwave) for the SNOWPACK surface layer, while sensible and latent heat fluxes are calculated by SNOWPACK and returned to WRF.

The SMB accounts for several components calculated by the surface is described by the equation:

3 $${SMB}=P+D+R-E-S-M$$

where P is the precipitation, D is deposition of ice particles to the ground, R is the refreeze of liquid water, E is the erosion of ice particles by the wind, S is the sublimation at the ground and M the ice melted to liquid water in mm. From these components, precipitation is the only component directly computed by WRF; sublimation, melt and refreeze are computed by the SNOWPACK surface model while erosion and deposition are exchanged by WRF and SNOWPACK via the blowing snow module.

### Physical processes of the heatwave

To study the changes in the physical atmospheric processes during the build-up and the duration of the heatwave, between 14 and 20 March 2022, we calculated the different components that govern the temperature and moisture tendency at CRYOWRF sigma levels in the atmosphere. These components are described through the equations:

4 $$\underbrace{\frac{\Delta T}{\Delta t}}_{{TendT}}=\underbrace{-\vec{v}\cdot {\nabla }_{p}T}_{{HAdvT}}+\underbrace{w\frac{\partial T}{\partial z}}_{{VAdvT}}+\underbrace{{Q}_{T}}_{{Diab}}$$

5 $$\underbrace{\frac{\Delta q}{\Delta t}}_{{TendQ}}=\underbrace{-\vec{v}\cdot {\nabla }_{p}q}_{{HAdvQ}}+\underbrace{w\frac{\partial q}{\partial z}}_{{VAdvQ}}+\underbrace{{Q}_{q}}_{{Source}/{Sink}}$$

where $T$ is the temperature, $t$ the time, $\vec{v}$ the horizontal wind, $w$ the vertical velocity, and $q$ the water vapor mixing ratio. The components of the equations. (4) and (5) represent the temperature and moisture tendency (*TendT* and *TendQ*), the horizontal temperature and moisture advection (*HAdvT* and *HAdvQ*), the vertical advection of temperature (or adiabatic heating) and moisture (*VAdvT* and *VAdvQ*) and the residual term that accounts mainly for the diabatic processes in the temperature equation (Diab) and local condensation or evaporation (Source/Sink) in the moisture equation. Notice that the last term also includes other non-accounted terms like the diffusive fluxes and computational errors, which can be further decomposed using Lagrangian methods^77^. For our purposes, we employ a conventional Eulerian decomposition, supposing that these non-accounted terms are of an order of magnitude lower; however, interpretation of residual values has to be taken with caution. This equation was evaluated at low levels, between the sigma level 2 and 5 (around 26 - 82 m over Concordia station at the top of Dome C). We solved the vertical derivative in the vertical advection terms by finite differences between the upper and lower layers.

### Firn air depletion, firn water content and ice lenses

Changes in firn were assessed using three different metrics: the FAC, the FWC and the pILT. The FAC and the FWC are described through the equations:

6 $${FAC}={\sum }_{i}^{N}{{\theta }_{a}}_{i}{\Delta z}_{i}$$

7 $${FWC}={\sum }_{i}^{N}{{\theta }_{w}}_{i}{\Delta z}_{i}$$

where ${{\theta }_{a}}_{i}$ is the volumetric air content and ${{\theta }_{w}}_{i}$ is the volumetric liquid water content of layer $i$, and ${\Delta z}_{i}$ is the thickness.

Since the simulation does not last long enough to refreeze all the water percolated, we define pILT as the total thickness of layers with pore close-off, defined as snow density ${\rho }_{s}$ > 830 kg m^−^^3^, so:

7 $${pILT}={\sum }_{i}^{N=100}{\Delta z}_{i}{for}{\rho }_{s} > 830{kg}{m}^{-3}$$

This includes pore close-off by liquid water and assumes that liquid water at the end of the event remains in the same layer and eventually refreezes, forming the ice lens. Since the snowpack was initialized with identical volumetric air, ice and water contents for every experiment, the evaluation for FAC and pITL focuses on changes at the end of the event on 25 March 2022. Due to the temporal changes in liquid water by melting and refreezing processes, FWC was evaluated as changes in the maximum FWC during the event.

### Reporting summary

Further information on research design is available in the Nature Portfolio Reporting Summary linked to this article.

## Supplementary information

Transparent Peer Review file

Supplementary material

Reporting summary

## Data Availability

ERA5 reanalysis data for CRYOWRF initialization and boundary conditions were obtained from the Copernicus Climate Data Store (https://cds.climate.copernicus.eu/). CMIP6 data used to calculate the PGW deltas were obtained from the Earth System Grid Federation (https://esgf-node.llnl.gov/projects/cmip6/). Concordia station data used to validate CRYOWRF was obtained from the Italian Antarctic Meteo-Climatological Observatory (https://www.climantartide.it/dataaccess/). PGW deltas generated for this study and CRYOWRF configuration files required to reproduce the experiments have been deposited at Envidat (10.16904/envidat.700). Due to their large size, full model outputs cannot be publicly uploaded, but can be reproduced with the provided input data. Selected subsets of the output are available from the corresponding author upon reasonable request for a prudential period of at least four years after publication.

## Code availability

CRYOWRF code can be accessed through https://gitlabext.wsl.ch/atmospheric-models/CRYOWRF. PGWERA5WRF to calculate the climate deltas and transform them to WRF intermediate files can be obtained at https://github.com/sergigonzalezh/PGWERA5WRF. The codes to reproduce the plots have been deposited at https://github.com/sergigonzalezh/2022AntarcticHeatwave_attribution_storylines.
