# Supplementary material for: Impact attribution of the March 2022 Antarctic heatwave reveals amplification by cloud feedbacks and increased future meltwater
Source: Commun Earth Environ. 2026 Apr 16;7(1):504. doi: 10.1038/s43247-026-03485-0 (PMC13259944; doi:10.1038/s43247-026-03485-0)
Supplement: Supplementary file 2 — Supplementary material [file 43247_2026_3485_MOESM2_ESM.pdf]

Impact attribution of the March 2022 Antarctic heatwave reveals amplification by cloud feedbacks and increased future meltwater

Sergi González-Herrero[1], Pranab Deb[2], Sihan Li[3], Daniel Argueso[4], Rainette Engbers[1,5], Michael Matějka[6], Nander Wever[1,7], Michael Lehning[1,5]

1WSL Institute for the Snow and Avalanche Research SLF, Davos Dorf, Switzerland (sergi.gonzalez@slf.ch)
2Indian Institute of Technology, Kharagpur, India
3University of Sheffield, Sheffield, UK
4Universitat de les Illes Balears, Palma, Spain
5EPFL, Sion, Switzerland
6Masarykova University, Brno, Czech Republic
7Meteoswiss, Zurich, Switzerland

1. Extended Figures

This section presents extended figures for the interpretation of the results.

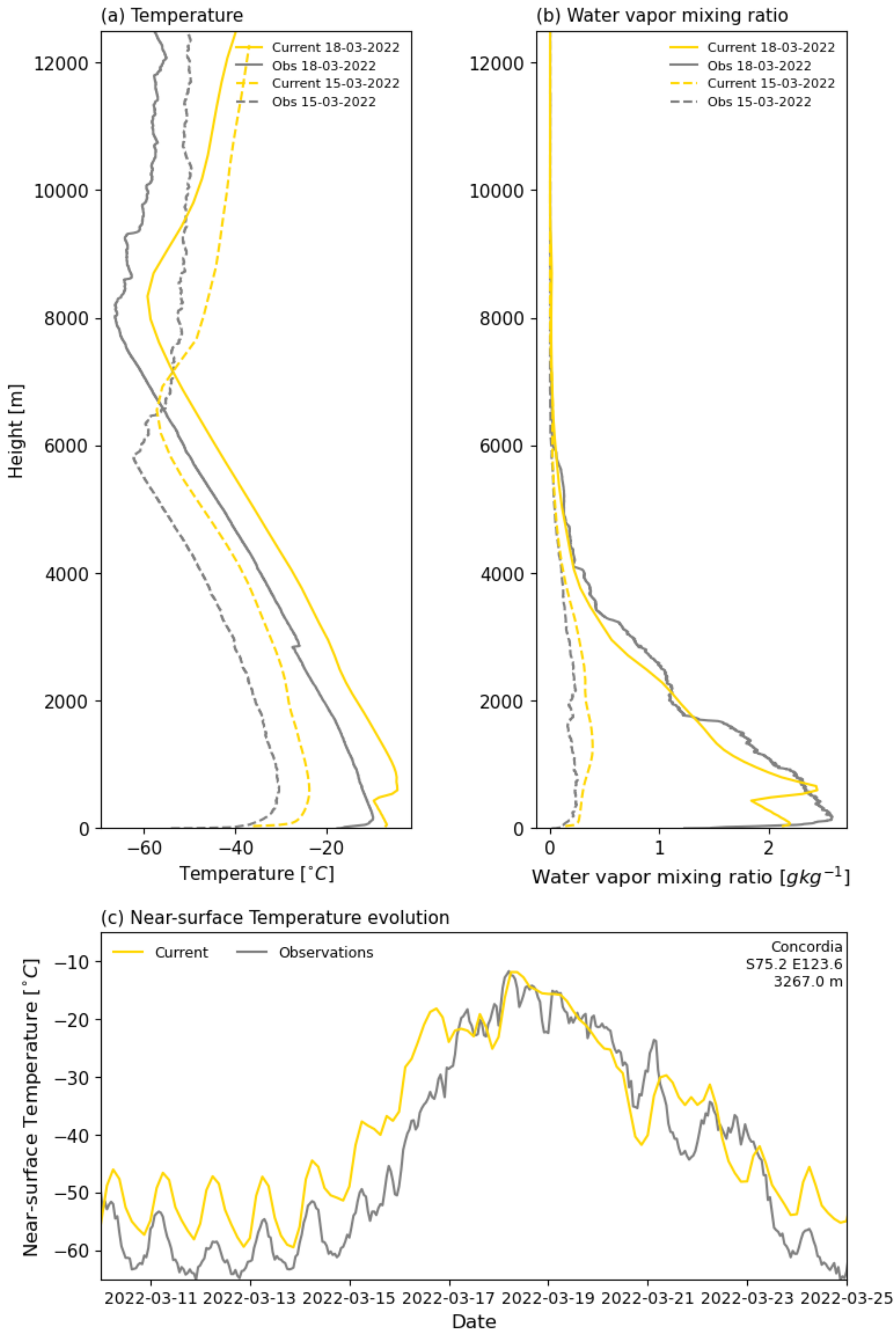

Supplementary Figure 1.1. Evaluation of the CRYOWRF current simulations. a Observed (grey lines) and simulated (yellow lines) temperature profiles on 15th March 2022 at 00 UTC (dashed lines) and on 18th March 2022 at 00 UTC (solid lines) over Concordia station (model coordinates S75.2 E123.6 at 3267m height). **b** Observed (grey lines) and simulated (yellow lines) water vapor mixing ratio profiles on 15th March 2022 at 00 UTC (dashed lines) and on 18th March 2022 at 00 UTC (solid lines) over Concordia station (model coordinates S75.2 E123.6 at 3267m height). **c** Temperature evolution observed (grey line) and simulated (yellow line) at the nearest grid point to Concordia station (model coordinates S75.2 E123.6 at 3267m height).

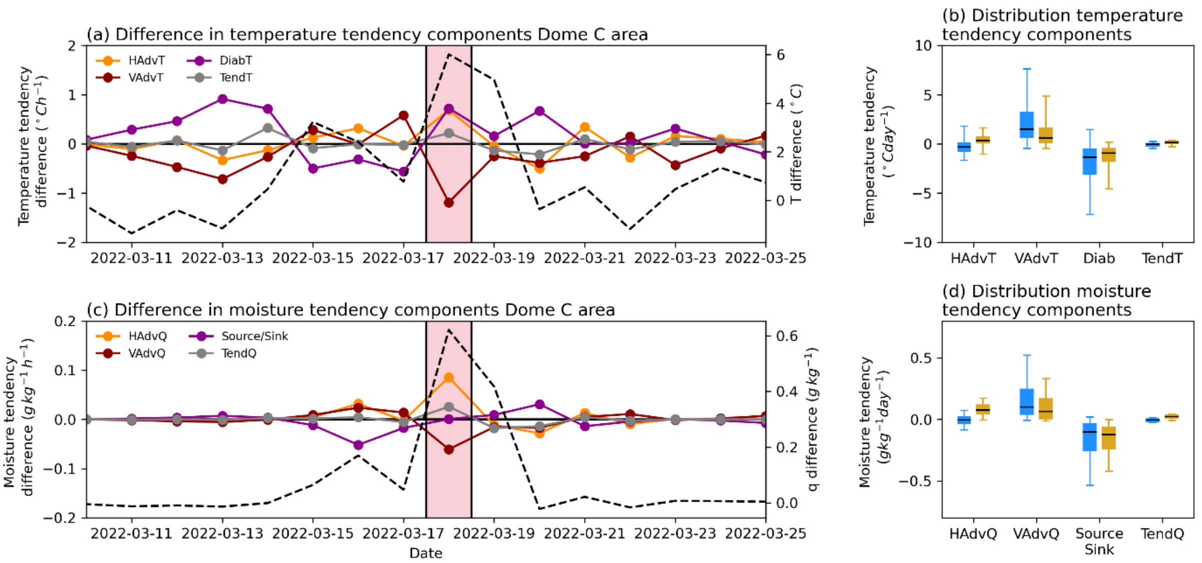

Supplementary Figure 1.2. Changes in temperature and moisture tendency components in Dome C region. a,c Timeseries of daily average of the difference in tendency equation components for the Dome C region between the Preindustrial and Current simulations, evaluated between the sigma layers 1 and 4 (~20 to 80 m a.g.l. over the dome). The pink area indicate the day of temperature divergence between current and Preindustrial simulation on 18th March 2022. The dashed black lines represents the mean temperature and moisture difference. **b,d** Distribution boxplots for each contributing factor in the Dome C region on 18th March 2022 for the preindustrial (blue) and current (yellow) simulations. The contributing terms include horizontal temperature and moisture advection (HAdvT, HAdvQ), the vertical temperature and moisture advection (VAdvT, VAdvQ) and diabatic heating or or sink/source terms (Diab; Source/Sink).

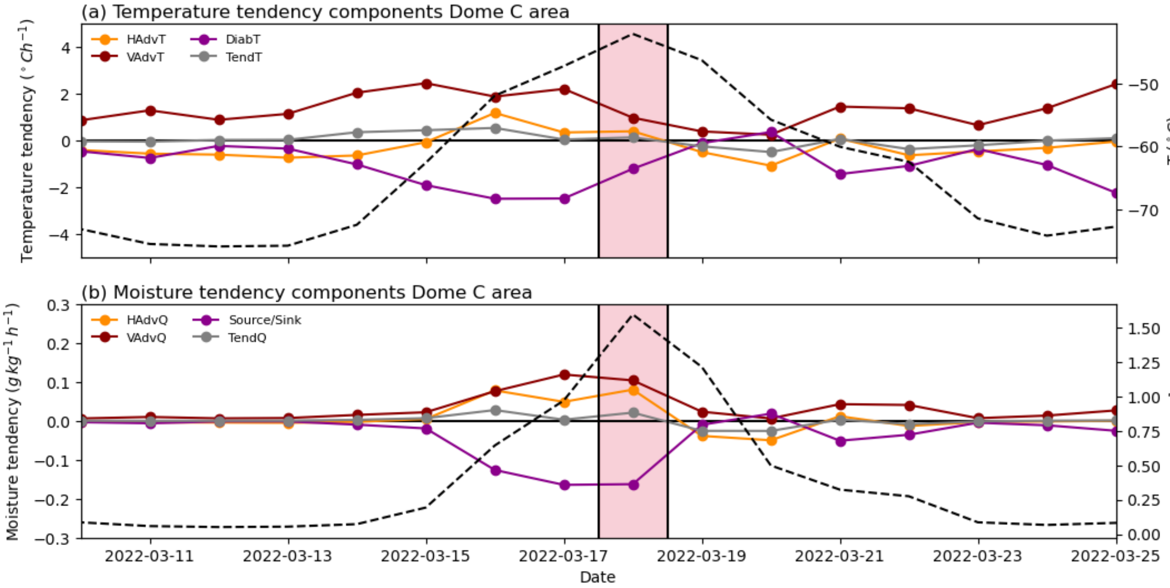

Supplementary Figure 1.3. Temperature and moisture tendency components in Dome C region in the current simulation. a,b Timeseries of daily average values in tendency equation components for the Dome C region, evaluated between the sigma layers 1 and 4 (~20 to 80 m a.g.l. over the dome). The pink area indicate the day of temperature divergence between current and Preindustrial simulation on 18th March 2022. The dashed black lines represent the mean temperature and moisture. The contributing terms include horizontal temperature and moisture advection (HAdvT, HAdvQ), the vertical temperature and moisture advection (VAdvT, VAdvQ) and diabatic heating or or sink/source terms (Diab; Source/Sink).

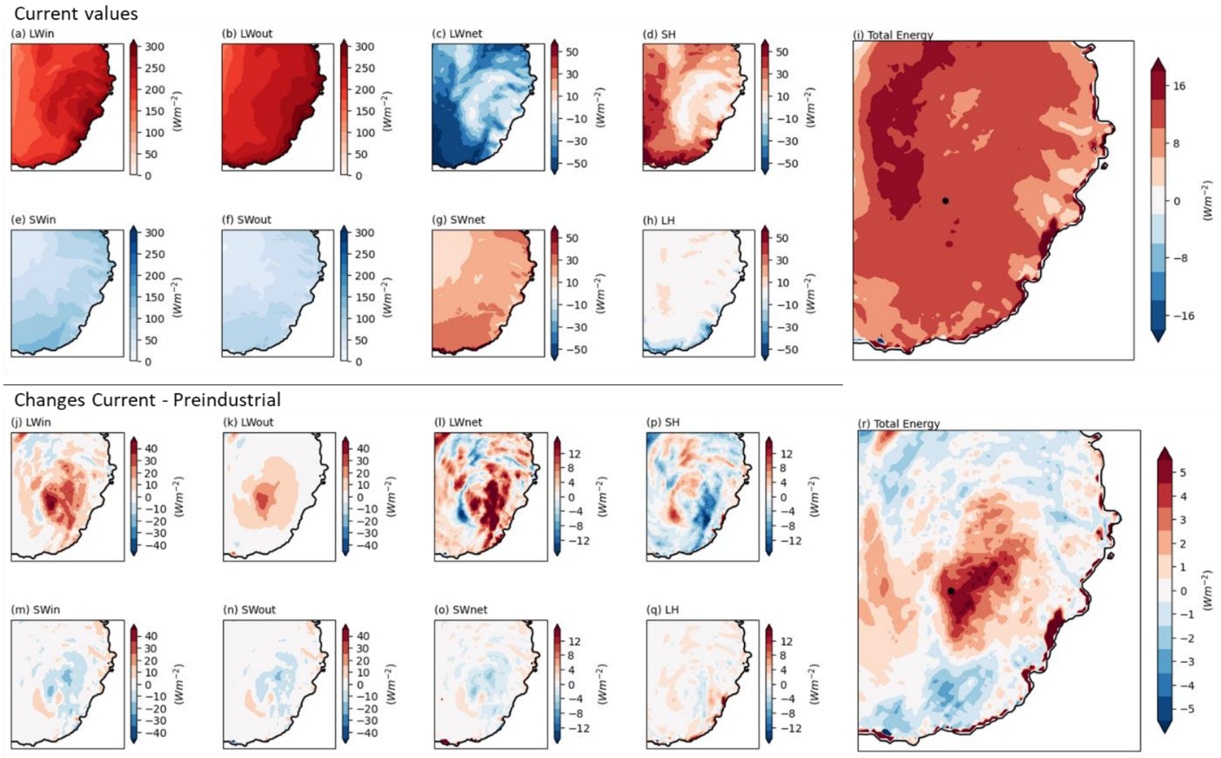

Supplementary Figure 1.4. Energy budget components and changes. a,b,c,d,j,k,l,m Incoming and outgoing components of the long wave (LW) and short wave (SW) radiation. **c,g** Net components of the LW and SW radiation. **d,h** Sensible (SH) and latent (LH) heat fluxes. **i** Sum of all the previous components. **a,b,c,d,e,f,g,h,i** Average components between 17-19 March 2022 in Wm^{-2} in the current simulation. **j,k,l,m,n,o,p,q,r** Changes in the average components between the preindustrial and current simulations.

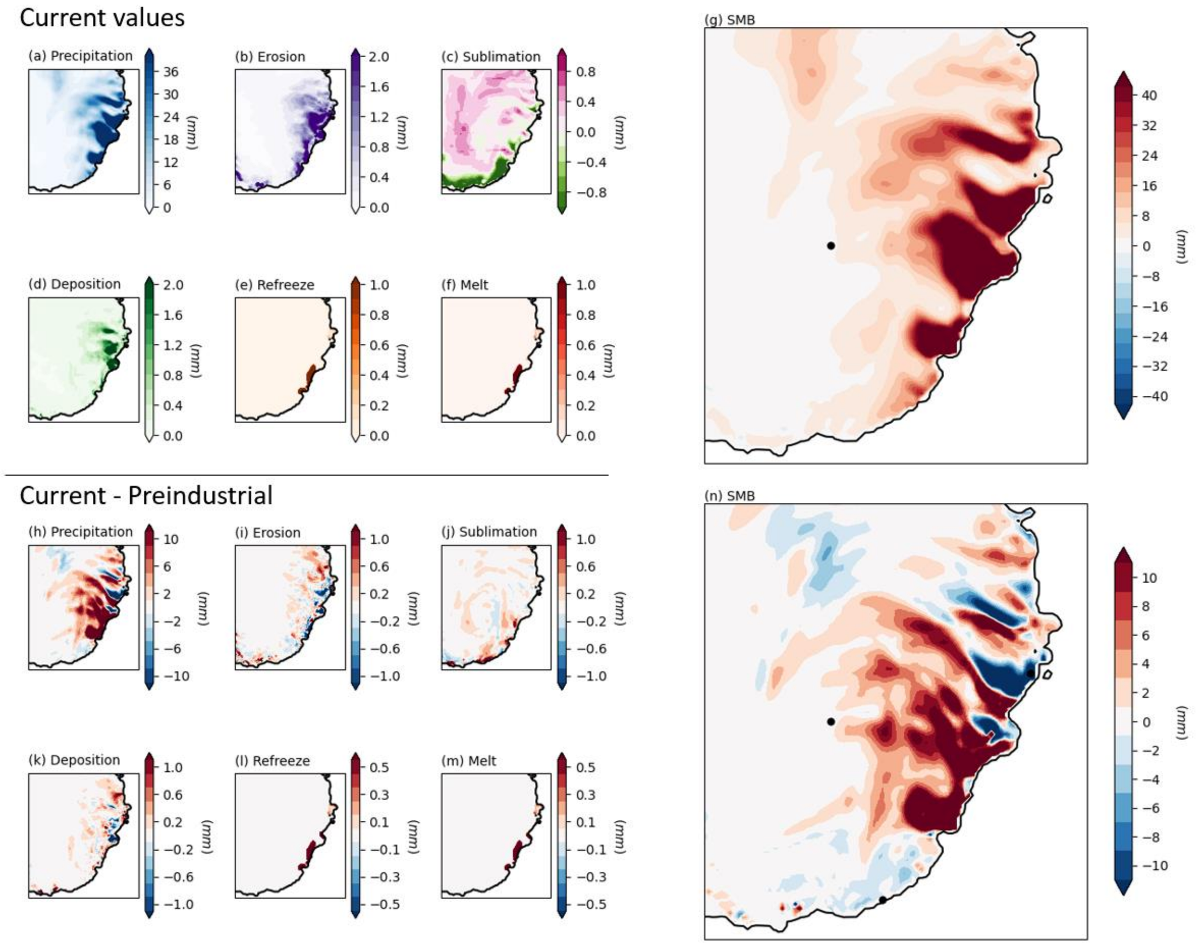

Supplementary Figure 1.5. SMB components and changes. a,b,c,d,e,f,g Variation in precipitation, erosion, sublimation, deposition, refreeze and melt between 17-19 March 2022 in mm. **h,I,j,k,l,m,n** Changes in the variation of the components between the preindustrial and current simulations.

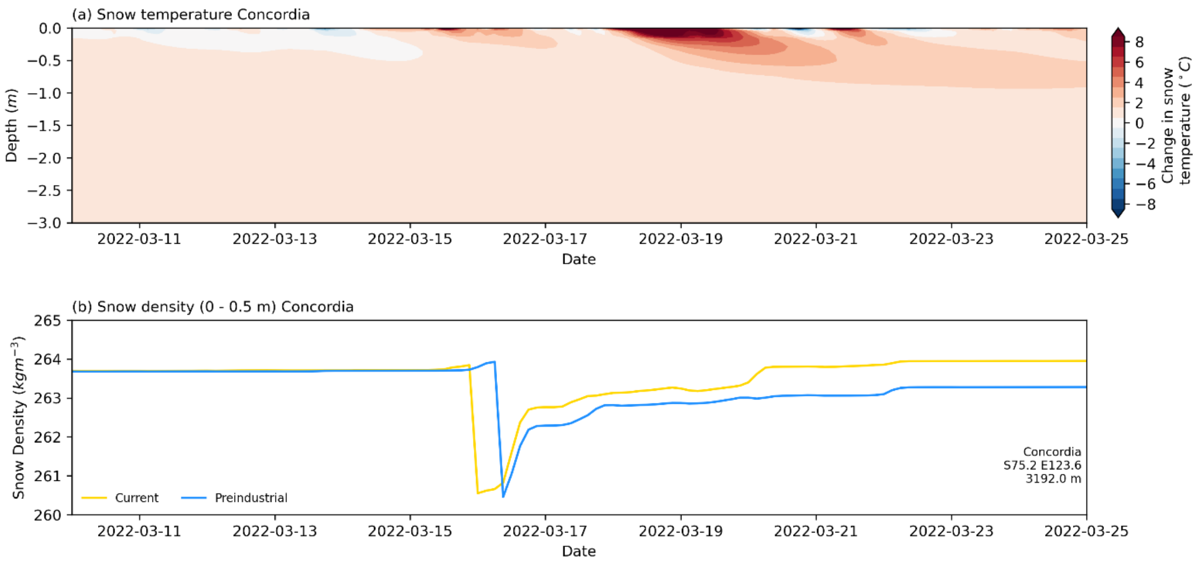

Supplementary figure 1.6. Snow and firn changes. a,b Changes in the surface snow temperature (a) and snow density of the first 0.5m (b) after the event at 25 March 2025. **a** Evolution of the snow temperature difference between the current and preindustrial simulation in the first 3 m at Concordia. **b** Evolution of the snow density of the first 0.5 m in the current and preindustrial simulations at Concordia.

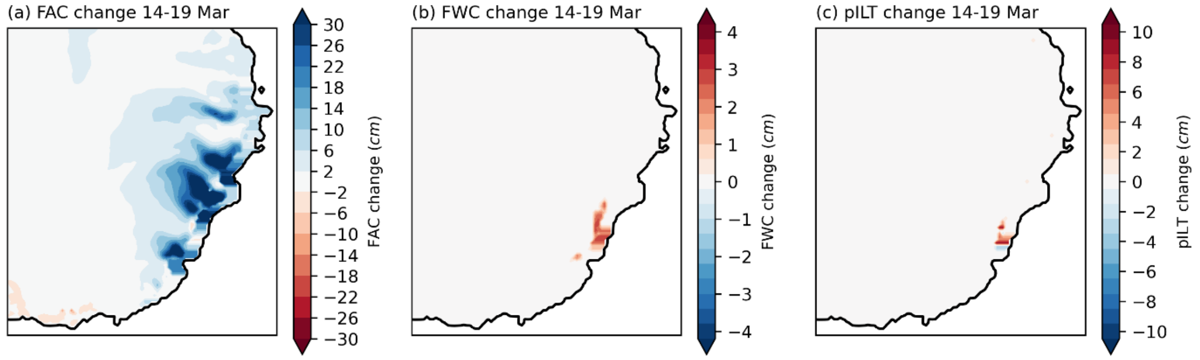

Supplementary Figure 1.7. Firn impact of the heatwave event in the current simulation. a Firn air content change, **b** firn liquid water content change and c potential ice lens thickness change between 14 and 19 March 2022.

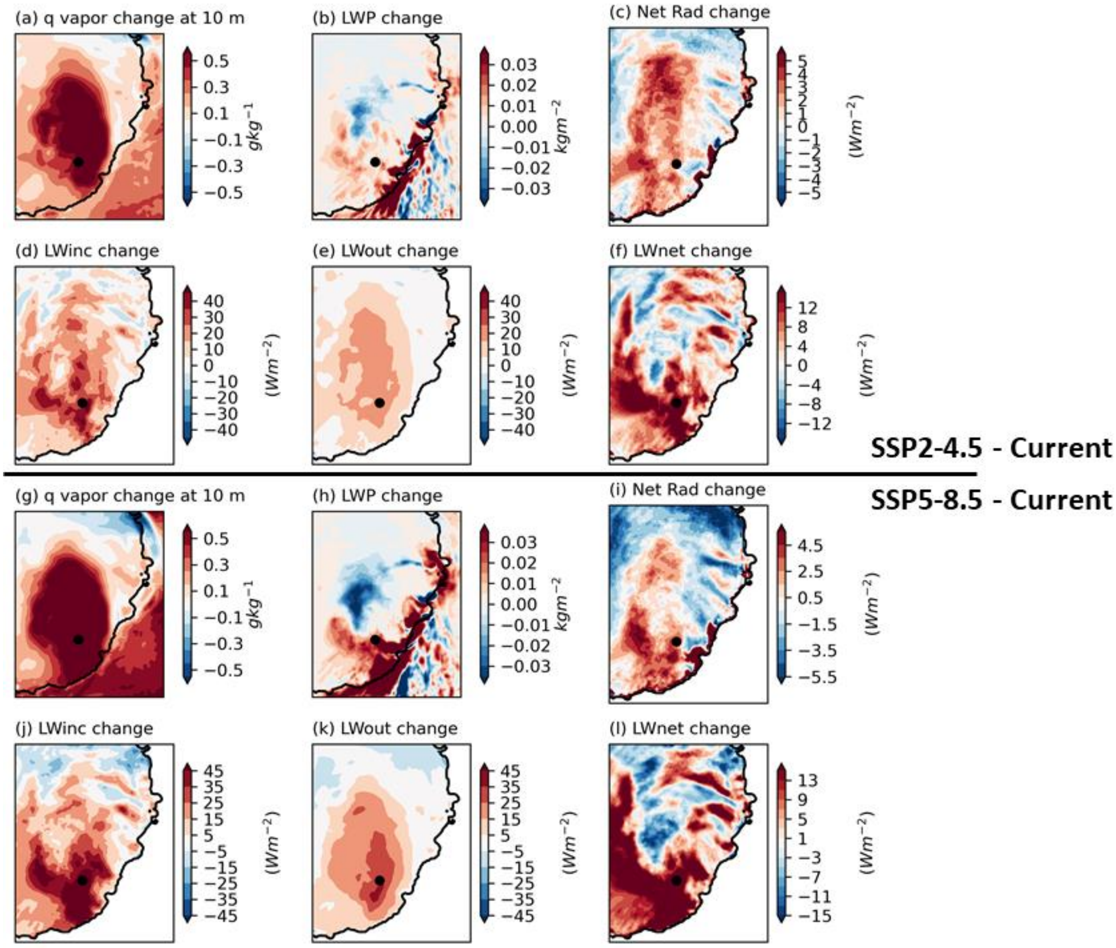

Supplementary Figure 1.8. Physical processes that contribute to the future temperature amplification by the end of the century. a,g,b,h Changes 17-19 March 2022 averaged vapor mixing ratio (q vapor in g kg^{-1}; a,g) and liquid water path (LWP in kg m^{-2}; b,h) between future and current simulations. **d,e,f,j,k,l** Changes in the 17-19 March 2022 averaged long wave (LW) incoming (d,j), outcoming(e,k) and net radiation (f,l) in Wm^{-2} between future and current simulations. **c,i** Changes in the 17-19 March 2022 net surface energy in Wm^{-2} between future and current simulations. **a,b,c,d,e,f** Changes between SSP2-4.5 by the end of the century and the current simulation. **g,h,l,j,k,l** Changes between SSP5-8.5 by the end of the century and the current simulation. In panels a,b,g,h, red (blue) colors represent an increase (decrease) of mixing ratio and LWP during the future simulation while in panels c,d,e,f,i,j,k,l, red (blue) colors represent an increase (decrease) of incoming radiation towards the surface.

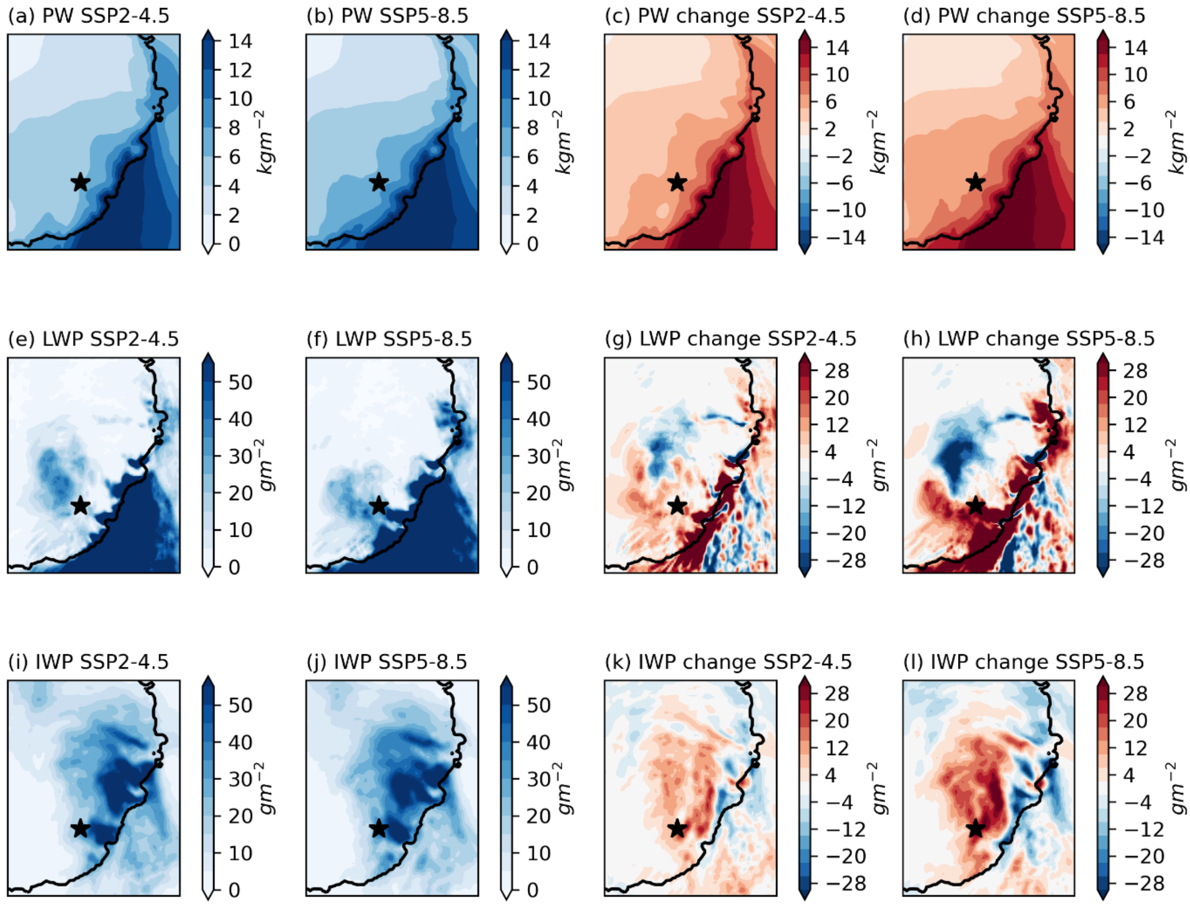

Supplementary Figure 1.9. Changes in vertically integrated water components in future simulations. a,b,c,d Precipitable water vapor (PW; kg m^{-2}), **e,f,g,h** liquid water path (LWP; g m^{-2}), and **i,j,k,l** ice water path (IWP; g m^{-2}), for the **a,e,i** SSP2-4.5 simulation, **b,f,j** SSP5-8.5 simulation, **c,g,k** change between SSP2-4.5 and current simulations and **d,h,l** change between SSP5-8.5 and current simulations. Values are averaged between 17-19 March 2022.

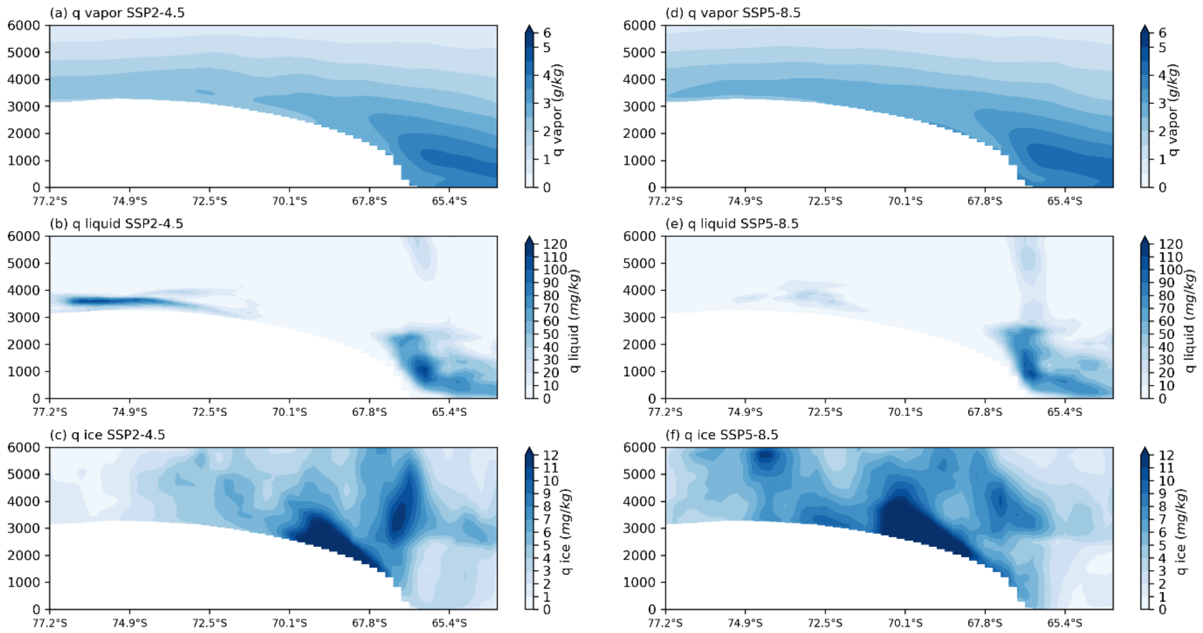

Supplementary Figure 1.10. Cross sections with the mixing ratio for future simulations. a,b Water vapor mixing ratio (qvapor; g kg^{-1}), **c,d** liquid water mixing ratio (qliquid; mg kg^{-1}), and **e,f** ice mixing ratio (qice; mg kg^{-1}), for the **a,c,e** SSP2-4.5 simulation, and **b,d,f** SSP5-8.5 simulation. Values are averaged between 17-19 March 2022.

2. Event evolution and values during the heatwave

This section presents the evolution of the event in the control (current-climate) simulation, with particular focus on the atmospheric water-related components: precipitable water (PW), liquid water path (LWP), and ice water path (IWP). Supplementary Figure 1.1 illustrates four key stages: the onset of the event (15 March), the peak intensity (18 March), the decay phase (20 March), and the aftermath (23 March).

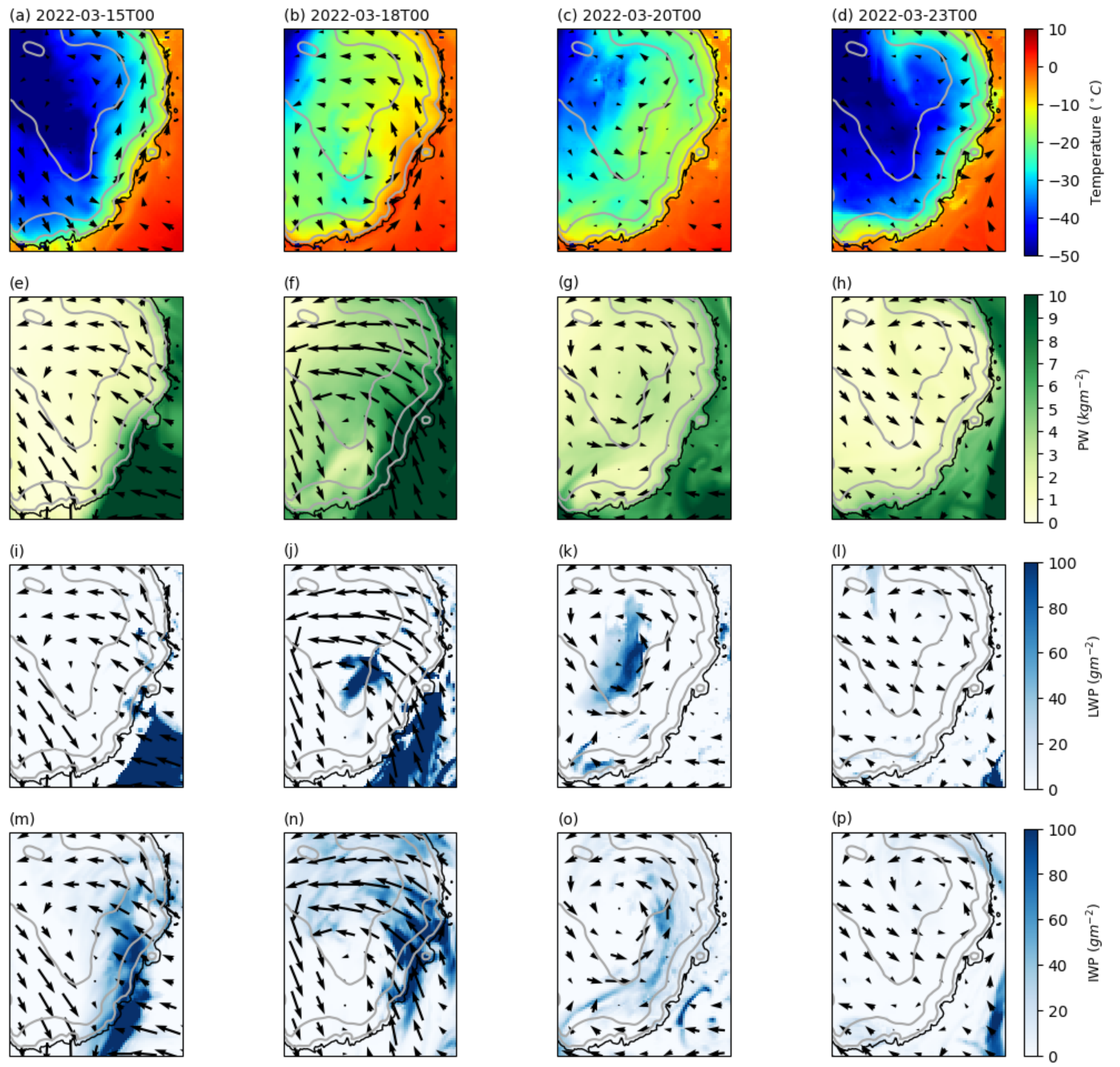

Supplementary Figure 2.1. Evolution of the atmospheric water components during the event. a-d 2m temperature (°C) and 10m wind speed and direction. **e-h** Precipitable water (kg m^{-2}) and 25th sigma level wind speed and direction. **i-l** Liquid water path (g m^{-2}) and 25th sigma level wind speed and direction. **m-p** Ice water path (g m^{-2}) and 25th sigma level wind speed and direction. Gray lines indicate the height contours every 1000 m. Figure shows 4 key stage of the event the onset (**a,e,i,m**), the peak (**b,f,j,n**), the decay (**c,g,k,o**), and the aftermath (**d,h,l,p**). 25th sigma level correspond to a height ca. 1843 m above Concordia station.

3. Amplification in percentage

Here we show Figs. 1 and 4 in percentage.

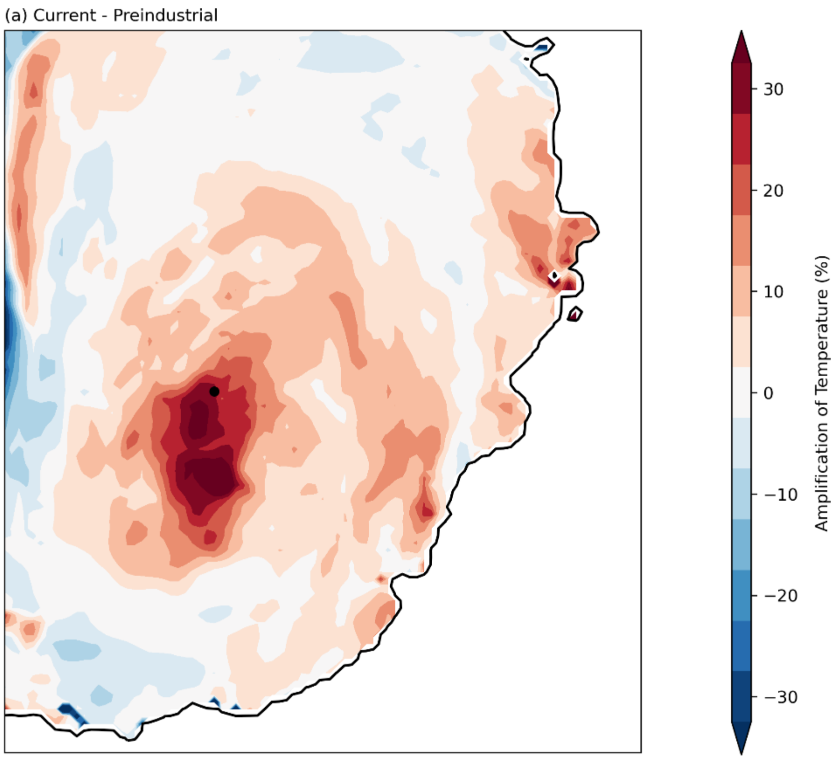

Supplementary Figure 3.1. Temperature amplification by climate change during 17-20 March 2022 in percentage. a As Fig. 1a but in percentage (%).

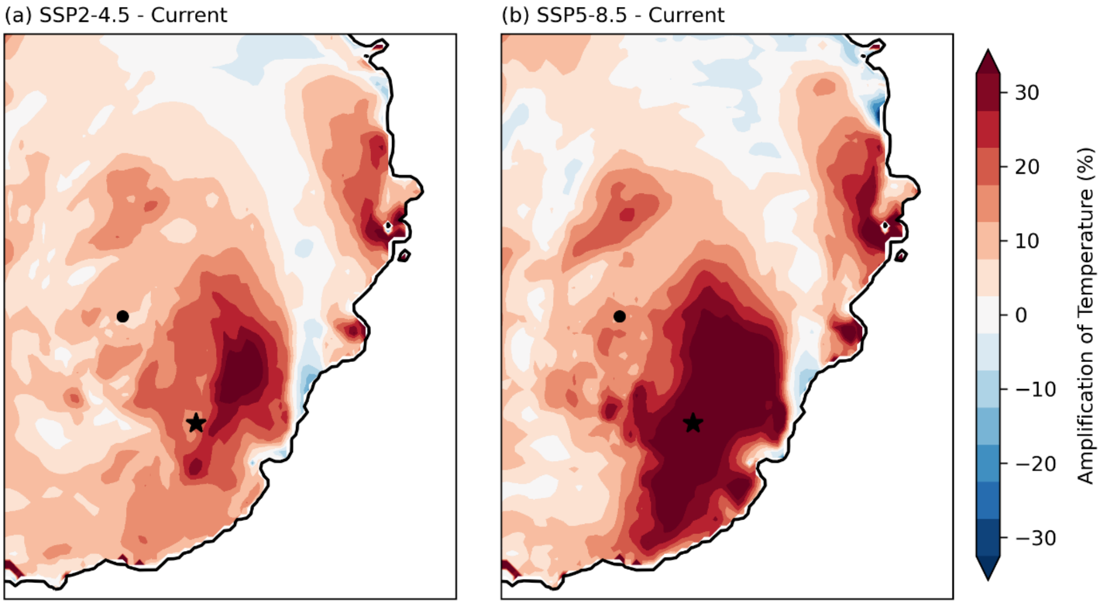

Supplementary Figure 3.2. Future scenarios for the event during 17-20 March 2022 by the end of the century in percentage. a,b As Fig. 5a and b but in percentage (%).

4. Pseudo-global warming approach

The approach followed in this study consists of modifying different variables as input and boundary conditions using the delta change method based on outputs from multiple Global Climate Models (GCMs). To reduce the influence of any single model, we used climatologies from six CMIP6 GCMs. Model selection was constrained by data availability, especially from polar-related variables like sea ice concentration, at the time when delta changes were calculated. Given the high computational cost of running CRYOWRF, we performed only one realization per experiment, applying the mean delta changes derived from the six selected GCMs. The CMIP6 models selected and the corresponding output variables that were modified are listed in the following table.

Supplementary Table 4.1. Global general circulation models and variables used to calculate climate deltas. The variables are written as the standard variable output name in the CMIP6 models.

CMIP6 models	Output variables
CAS-ESM2-0	Relative humidity (hur)
CMCC-CM2-SR5	Near-surface relative humidity (hurs)
CMCC-ESM2	Surface air pressure (ps)
EC_Earth3	Mean sea level pressure (psl)
FGOALS-f3-L	Sea Ice Concentration (siconc)
MRI-ESM2-0	Air temperature (ta)
	Near-surface air temperature (tas)
	Sea surface temperature (tos)
	Surface temperature (ts)
	Eastward wind (ua)
	Eastward near-surface wind (uas)
	Northward wind (va)
	Northward near-surface wind (vas)
	Geopotential height (zg)

Variable	Experiment delta	CAS-ESM2-0	CMCC-CM2-SR5	CMCC-ESM2	EC_Earth3	FGOALS-f3-L	MRI-ESM2-0
2m temperature (°C)	current - preindustrial	0.81	1.30	1.09	1.02	1.46	1.25
	SSP2-4.5-current	2.20	2.17	2.75	1.96	1.84	2.57
	SSP5-8.5-current	4.31	4.45	4.39	3.46	3.56	4.63
2m relative humidity (%)	current - preindustrial	-0.4	-1.2	-0.9	0.2	-0.6	-0.8
	SSP2-4.5-current	-1.3	-1.5	-1.9	0.8	0.1	-0.9
	SSP5-8.5-current	-2.0	-3.0	-3.0	-1.7	0.2	-1.7
Sea surface temperature (°C)	current - preindustrial	0.25	0.34	0.32	0.28	0.41	0.27
	SSP2-4.5-current	1.02	1.21	1.21	1.04	0.88	0.99
	SSP5-8.5-current	1.66	1.90	1.89	1.84	1.63	1.69

Supplementary Table 4.2. Climate deltas for 2m temperature, 2m relative humidity and sea surface temperature for the different CMIP6 models used in this study.

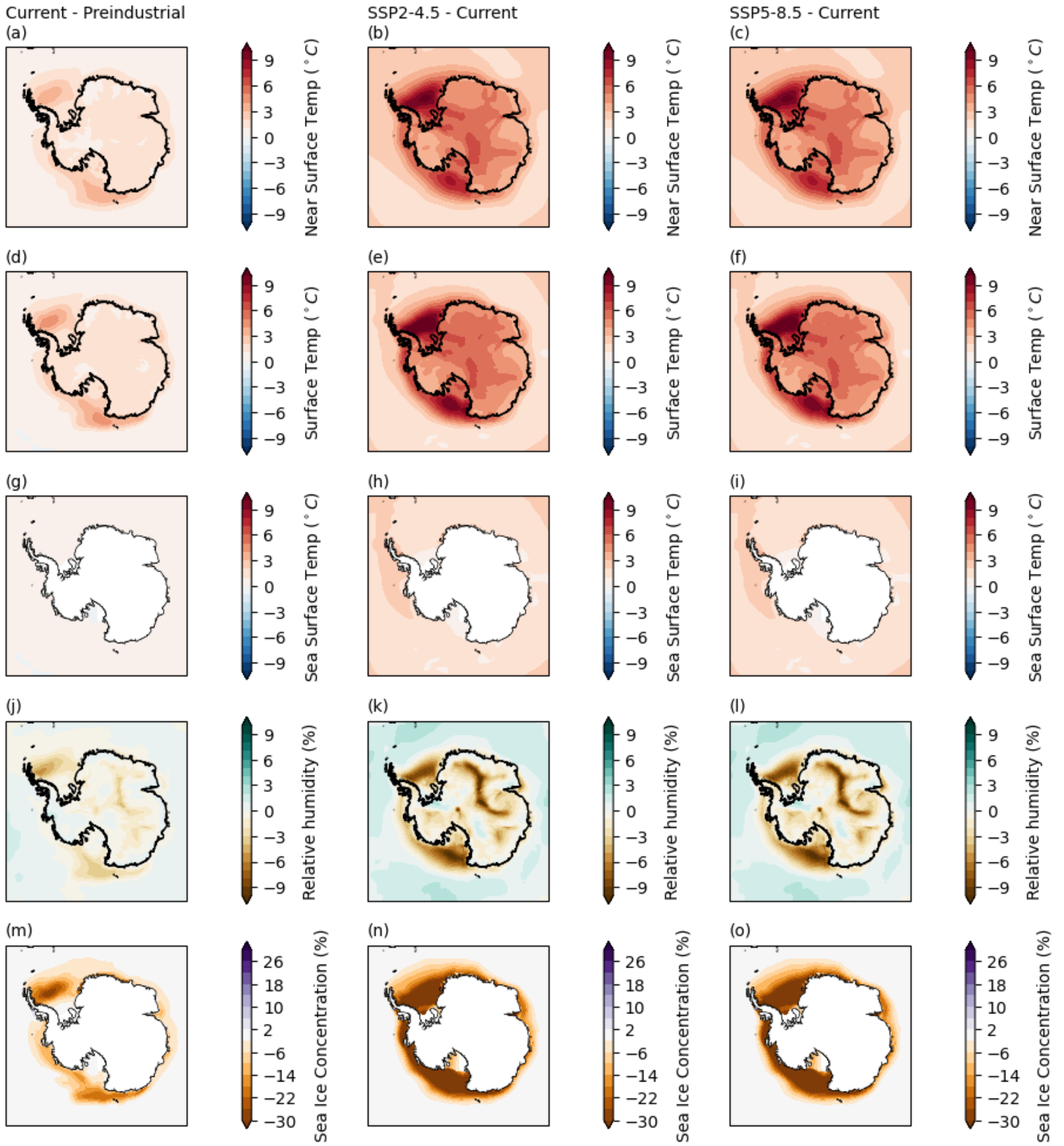

Supplementary Figure 4.1. Average delta components for March from the six CMIP6 models used to modify initial and boundary conditions for our simulations. a,b,c Near-surface temperature (tas in °C), **d,e,f** Surface temperature (ts in °C), **g,h,i** Sea surface temperature (tos in °C), **j,k,l** Relative humidity (hurs in %), **m,n,o** Sea ice concentration (siconc in %). **a,d,g,j,m** Changes between current and preindustrial simulations, **b,e,h,k,n** Changes between the SSP2-4.5 and current simulations, **c,f,i,l,o** Changes between the SSP5-8.5 and current simulations.
