# Supplementary material for: Impact attribution of the March 2022 Antarctic heatwave reveals amplification by cloud feedbacks and increased future meltwater
Source: Commun Earth Environ. 2026 Apr 16;7(1):504. doi: 10.1038/s43247-026-03485-0 (PMC13259944; doi:10.1038/s43247-026-03485-0)
Supplement: Supplementary file 3 — Reporting summary [file 43247_2026_3485_MOESM3_ESM.pdf]

Permission

SPRINGER NATURE

The Author(s) Sergi González-Herrero and coauthors (**"Author(s)"**) request permission from the Rightsholder Sergi González-Herrero (**"Rightsholder" / "You"**), to publish the source material, outlined below as part of a work written for an academic/professional audience, provisionally titled Impact attribution of the March 2022 Antarctic heatwave reveals amplification by cloud feedbacks and increased future meltwater (**"Work"**), to be published by Springer Nature.

Source Material:
Title or description of source material: Photography of Antarctic Clouds
Author/Creator: Sergi González-Herrero
Excerpt type: ☐ Text ☒ Illustration/figure ☐ Image/Photograph ☐ Other: Click here to enter text
Other details (if applicable e.g. DOI, ISBN, Page Number, copyright year): Click here to enter text
(the "**Material**")

For good and valuable consideration, you agree to the following terms and conditions:
You grant the Author(s) and the current and future Springer Nature group companies and their licensees a non-exclusive, worldwide, royalty free right and licence (i) to publish, reproduce, display, and distribute the Material within the Work and all related editions, revisions, translations and for the full period of copyright and in all media whether now known or hereafter devised; (ii) the right to modify the Material for stylistic purposes, and (iii) use the Material for marketing and promotional material (including without limitation on third party social media sites) but at all times in relation to the Work (the "Rights"). Nothing in this permission creates any obligation to publish the Material. The Rightsholder will be acknowledged in the Work.

Open Access
*This section only applies where the **Author(s)** are publishing their **Work** under an Open Access Licence and have indicated this by ticking the following box:*
☒ The Work will be published Open Access under a CC BY licence.

Rightsholder to indicate their preference for the Material
☒ You agree to the **Material** being licenced to readers under the same CC BY licence as the Work
☐ You do **not** agree to the **Material** being licensed to readers under the same CC BY licence as the Work; the **Material** will be excluded from the Creative Commons licence and marked accordingly.

CC BY Licence Terms
Creative Commons licence CC BY 4.0: This licence allows readers to copy, distribute and transmit the Material as long as it is attributed back to the Rightsholder. Readers are permitted to alter, transform or build upon the Material, and to use the Material for commercial purposes. Please read the full licence for further details at http://creativecommons.org/licenses/by/4.0/
The Author(s) and Springer Nature may also choose to publish the Work under one of the more restrictive Creative Commons licences (-NC, -ND, -SA), but never a broader one.

Please note that even if not currently planned, the Author(s) and Springer Nature may in future make the Work available under the CC BY Creative Commons licence or one of the more restrictive licences (-NC, -ND, -SA); in this case the Material will be excluded from the Creative Commons licence and be marked accordingly.
You warrant and represent that you have full authority to grant this permission and are the sole owner of all the Rights in the Material or, in the event that you are not the sole owner, you have obtained all necessary permissions and/or licences to grant the Rights; and that the Material does not infringe the copyright or any other rights of any third party, including any right of confidentiality or privacy. You also warrant and represent that you have obtained any necessary release(s), from identifiable individuals and property/location owners. You agree to supply copies of any permissions, licences and/or releases to the Author(s) if requested.

The Rightsholder agrees to the above terms and conditions by signing and returning this permission

Signature: ______________________________ Date: 13.03.2026

Address: Davos, Switzerland

Please keep this copy for your records
