# Supplementary material for: Impact attribution of the March 2022 Antarctic heatwave reveals amplification by cloud feedbacks and increased future meltwater
Source: Commun Earth Environ. 2026 Apr 16;7(1):504. doi: 10.1038/s43247-026-03485-0 (PMC13259944; doi:10.1038/s43247-026-03485-0)
Supplement: Supplementary file 1 — Transparent Peer Review file [file 43247_2026_3485_MOESM1_ESM.pdf]

nature portfolio

# Peer Review File

# Impact attribution of the March 2022 Antarctic heatwave reveals amplification by cloud feedbacks and increased future meltwater

Corresponding Author: Dr Sergi González-Herrero

**This file contains all editorial decision letters in order by version, followed by all author rebuttals in order by version.**

Version 0:

Decision Letter:

** Please ensure you delete the link to your author home page in this e-mail if you wish to forward it to your coauthors **

Dear Dr González-Herrero,

Your manuscript titled "Impacts of Antarctic heatwaves amplified by climate change through water vapor and cloud feedbacks" has now been seen by 2 reviewers, and we include their comments at the end of this message. They find your work of interest, but some important points are raised. We are interested in the possibility of publishing your study in Communications Earth & Environment, but would like to consider your responses to these concerns and assess a revised manuscript before we make a final decision on publication.

We therefore invite you to revise and resubmit your manuscript, along with a point-by-point response that takes into account the points raised. Please highlight all changes in the manuscript text file.

Please submit your point-by-point responses as a separate file, distinct from your cover letter where you can add responses to the Editors' comments that you do not want to be made available to the reviewers. Word files are preferred. We recommend that any figures, tables or graphs that are included in the response to reviewers are also included in the main article or Supplementary Information.

We are committed to providing a fair and constructive peer-review process. Please don't hesitate to contact us if you wish to discuss the revision in more detail.

Please use the following link to submit your revised manuscript, point-by-point response to the referees' comments (which should be in a separate document to any cover letter), a tracked-changes version of the manuscript (as a PDF file) and the completed checklist:
Link Redacted
** This url links to your confidential home page and associated information about manuscripts you may have submitted or be reviewing for us. If you wish to forward this email to co-authors, please delete the link to your homepage first **

We hope to receive your revised paper within six weeks; please let us know if you aren't able to submit it within this time so that we can discuss how best to proceed. If we don't hear from you, and the revision process takes significantly longer, we may close your file. In this event, we will still be happy to reconsider your paper at a later date, as long as nothing similar has been accepted for publication at Communications Earth & Environment or published elsewhere in the meantime.

Please do not hesitate to contact us if you have any questions or would like to discuss these revisions further. We look forward to seeing the revised manuscript and thank you for the opportunity to review your work.

Best regards,

Haoyu Jiang, PhD
Editorial Board Member
Communications Earth & Environment

ChenRui Diao, PhD
Associate Editor,
Communications Earth & Environment
Consulting Editor,
Communications Sustainability

EDITORIAL POLICIES AND FORMATTING

We ask that you ensure your manuscript complies with our editorial policies. Please ensure that the following formatting requirements are met, and any checklist relevant to your research is completed and uploaded as a Related Manuscript file type with the revised article.

For Manuscripts that fall into the following fields:
• Behavioural and social science
• Ecological, evolutionary & environmental sciences
• Life sciences
An updated and completed version of our Reporting Summary must be uploaded with the revised manuscript
You can download the form here:
https://www.nature.com/documents/nr-reporting-summary.pdf

Furthermore, please align your manuscript with our format requirements, which are summarized on the following checklist: <a href="https://www.nature.com/documents/commsj-phys-style-formatting-checklist-article.pdf">Communications Earth & Environment formatting checklist</a>

and also in our style and formatting guide <a href="https://www.nature.com/documents/commsj-phys-style-formatting-guide-accept.pdf">Communications Earth & Environment formatting guide</a> .

*** DATA: Communications Earth & Environment endorses the principles of the Enabling FAIR data project (http://www.copdess.org/enabling-fair-data-project/ ). We ask authors to make the data that support their conclusions available in permanent, publically accessible data repositories. (Please contact the editor if you are unable to make your data available).

All Communications Earth & Environment manuscripts must include a section titled "Data Availability" at the end of the Methods section or main text (if no Methods). More information on this policy, is available at <a href="http://www.nature.com/authors/policies/data/data-availability-statements-data-citations.pdf">http://www.nature.com/authors/policies/data/data-availability-statements-data-citations.pdf</a>.

In particular, the Data availability statement should include:
- Unique identifiers (such as DOIs and hyperlinks for datasets in public repositories)
- Accession codes where appropriate
- If applicable, a statement regarding data available with restrictions
- If a dataset has a Digital Object Identifier (DOI) as its unique identifier, we strongly encourage including this in the Reference list and citing the dataset in the Data Availability Statement.

DATA SOURCES: All new data associated with the paper should be placed in a persistent repository where they can be freely and enduringly accessed. We recommend submitting the data to discipline-specific, community-recognized repositories, where possible and a list of recommended repositories is provided at <a href="http://www.nature.com/sdata/policies/repositories">http://www.nature.com/sdata/policies/repositories</a>.

If a community resource is unavailable, data can be submitted to generalist repositories such as <a href="https://figshare.com/">figshare</a> or <a href="http://datadryad.org/">Dryad Digital Repository</a>. Please provide a unique identifier for the data (for example a DOI or a permanent URL) in the data availability statement, if possible. If the repository does not provide identifiers, we encourage authors to supply the search terms that will return the data. For data that have been obtained from publically available sources, please provide a URL and the specific data product name in the data availability statement. Data with a DOI should be further cited in the methods reference section.

Please refer to our data policies at <a href="http://www.nature.com/authors/policies/availability.html">http://www.nature.com/authors/policies/availability.html</a>.

REVIEWER COMMENTS:

Reviewer #2 (Remarks to the Author):

Review of “Impacts of Antarctic heatwaves amplified by climate change through water vapor and cloud feedbacks” by González-Herrero et al., 2025

This paper investigates the influence of climate change on an Antarctic heatwave event that occurred in 2022, using a high-resolution coupled snow-atmosphere model. They find that water vapor radiative effects and cloud feedbacks amplify the near-surface warming in the present climate relative to the preindustrial, with implications for the regional surface mass balance. This is an interesting paper that explores feedbacks and processes that may be underrepresented in coarser GCMs. However, I have concerns about some of the analyses, particularly the heat and moisture budgets. I also think some of the figures could be improved and condensed, and that the authors should better address the differences in lower-level structure between their simulations and the observations (Extended Figure 1). For these reason I suggest this paper undergo major revisions.

Major comments (scientific):
#1. The budgets for temperature and moisture tendencies are performed at different levels – sigma level 25 for temperature (~1800m) and sigma level 2 for moisture (~50m). The authors state in the methods that the temperature tendency analysis is conducted at a higher level to "assess processes above the atmospheric boundary layer," while the moisture tendency is done near the surface to examine "near-surface advection of moisture." However, since atmospheric rivers transport both moisture and heat together in the lower levels of the troposphere, the difference in height between the analyses is unintuitive, and also makes it difficult to compare the magnitudes of the effects. Given that the event being studied is a near-surface level temperature anomaly, it would make more sense to do budgets for both variables closer to the surface for consistency.

#2. In both the temperature tendency budget and the surface energy flux budget, the influence of horizontal/vertical sensible heat flux is not discussed in the text, despite being quite high in magnitude (Extended Figure 2, Supplementary Figure 2.1). ARs transport large amounts of sensible heat and cause anomalous downwards sensible heat flux at the surface, which is part of why they can cause such extreme surface temperature anomalies (e.g., Scholz & Lora, 2024). There are also obvious links between the changes in horizontal sensible heat transport and the surface flux changes that are not discussed. These results require further discussion in the text.

#3. As shown in Extended Figure 1, the simulations are warmer and have a different lower-level structure than observations, particularly the height of the inversion. This seems a substantial discrepancy to me, especially when considering the influence of this inversion height on the cloud processes which are invoked in the paper. Further justification of these differences is needed.

#4. Some assessment or measure of the statistical significance of results is needed. Particularly when the changes between experiments are only minimal, it would be useful to know how significant (or not) those changes are.

Major comments (figures/formatting):
#1. Maps should have latitude/longitude to orient the viewer. It may also be helpful to add topographic contours to Figure 1, or to have a label or shading that shows Dome C for reference.

#2. There are additional figures presented as both "Extended Figures" and "Supplemental Figures." It is not clear to me what criteria was used to decide which figures go in which category, as often the supplemental figures are cited more in the text that the extended data figures. I would suggest putting all the figures most relevant to the main text in the extended data if possible. In addition, some supplemental/extended figures that are not referenced or relevant to the main text could be removed or condensed to ease interpretation.

Line comments:

L42. 25% of the event's magnitude – how much of an amplification is this, compared to the value you find?

L47. Similarly, how much amplification does this 2C figure represent?

L49. A brief description of what the analog approach vs. the storyline approach are would be helpful here, before the limitations of both methods are discussed.

L66. 'create detailed storylines' – please clarify what this means in the context of the model

L81. Label Dome C on map.

L95-97. This sentence is unclear, what is meant by "no amplification under current climate conditions in altitude?"

L98. "No dominant driver above the boundary layer" but there is substantial horizontal sensible heat advection, though this seems to be somewhat balanced by diabatic cooling (further explanation of this would be helpful too). I do not think we can conclude from these figures that there is no higher-level influence on the warm surface temperatures, and it certainly does not exclude the role of sensible heat flux either.

L110-112. This argument is not very clear, and the feature mentioned is not obvious in the plots referenced.

L119. 'Removing energy… on the windward side:' this is not exactly accurate, as the figure shows the difference between the preindustrial and current, so the negative values just represent 'less downward' sensible heat flux. Also, this needs a reference to the appropriate figure number.

L126. This is the only time that Figure 3 appears to be cited, and only 3 components of it are mentioned.

L127-128. Are there relevant topographic reasons why this is occurring?

L129. Clarify here that you are referring to the exact proportion of LWP vs IWP, etc., otherwise it seems to contradict the budget analysis. Additionally, given the changes in LWP/IWP/etc., isn't there a way to estimate the estimate the longwave contribution of these different factors?

L131. 13-43 g m-2 of supercooled water?

L135. This doesn't exactly confirm it, but provides some strong evidence suggesting that it is a plausible cause.

L151. "redistribution between the two experiments" – unclear, please clarify.

L158. Why does the density remain lower in the preindustrial simulation, and what implications does this have?

L181. This argument is unclear and needs further explanation, please clarify. In addition, wouldn't a warmer world lead to a change in the 'physical limit' for supercooled water effects over the dome?

L185. More details about why firn air content is a relevant or important metric would be helpful here.

L199. Why are liquid-bearing clouds less prevalent than in the Arctic?

L207-210. Yes, the peak temperature is closer, but the CRYOWRF simulations are also several degrees hotter in general, which makes this statement less convincing. See major comment #3.

L213. The impact of the hydrostatic approximation in GCMs is mentioned several times in the intro and conclusion. However, the exact influence of using a non-hydrostatic scheme in this model is not explicitly stated; how would the results be different if hydrostasy was assumed? The vertical motion of sensible heat seemed to play a minimal role of the budget, for example.

L217. The link between ARs and supercooled liquid water clouds is not clear.

L244-246. Run on sentence, please rephrase for clarity.

L247. This argument seems contradictory - in L225, you state that CMIP6 models overestimate the occurrence of these clouds, but here you state that the amplification due to these clouds is largely absent from current GCMs? Please clarify.

L305-307. Given that this paper focuses on water vapor and cloud feedbacks, this seems to be a critical difference. See major comment #3.

L353. See major comment #1 about the temperature and moisture budgets.

L358. In addition to the horizontal and vertical advection, local convergence of both sensible heat and moisture are important in the lower-level dynamics of ARs, playing a role in warming the surface.

L365. The residual "Diab" term here could include a large variety of effects, which may not be appropriate to group together. I would consider this breakdown more carefully. Adiabatic and diabatic effects can also be estimated separately (e.g. Röthlisberger & Papritz, 2023).

L373. These components at different vertical levels are not shown or referenced.

Extended Figure 4. In both this figure and Figure 6, the changes in FWC and pILT are very small. Further information on why these spatially-limited changes are relevant would help make their importance more clear.

Supplement: The figure showing changes in temperature tendency from preindustrial to current (Supplementary Figure 2.1) appears to be very noisy – why is this?

References:
Scholz SR, Lora JM. Atmospheric rivers cause warm winters and extreme heat events. Nature. 2024 Dec;636(8043):640–6.
Röthlisberger M, Papritz L. Quantifying the physical processes leading to atmospheric hot extremes at a global scale. Nat Geosci. 2023 Mar;16(3):210–6.

Reviewer #3 (Remarks to the Author):

The manuscript " Impacts of Antarctic heatwaves amplified by climate change through water vapor and cloud feedbacks " investigates the climate change contribution to the March 2022 East Antarctic heatwave using pseudo-global warming

experiments with the snow-atmospheric coupled model CRYOWRF. It is significant due to land ice loss under global warming, however, the manuscript is should make major revisions as below.
1) Figure 2. b-c is vertical profile in lower troposphere atmosphere? How to understand column axis unit?
2) How to define near-surface? Why not use 2m temperature?
3) In abstract:"linearly amplify near-surface warming by up to 25% relative to preindustrial conditions", how to get this 25%? In the second paragraphs in main, "representing around 25% of the events magnitude, Are there other other contributions such as adiabatic heating due to descending motion?
4) Fig 3. liquid water path over observation station seems an independent and is far from ocean, how to link with sources of liquid water?
5) Some possible drivers should be discussed that warming further intensifies this amplification of near-surface warming.
6) Previous studies shows ice cloud contribute warm, however water vapor is also a kind of greenhouse gases, In Fig.3, you talk about ice water path and liquid water path, can you shows the contributions of them to near-surface temperature?
7) Figure 4 SMB seems the result of near-surface temperature or global warming forcing on surface energy and SMB, it is far from topic, I suggest to delete it.
8) Fig.5, near surface temperature is so lower, how to define heatwave of positive temperature in 2022 ? Is it suitable?
9) Antarctic warming effect is emphasized.Recommend a manuscript "Zhang J.*, H. Chen, X. Fang, Z. Yin, R. Hu. Warming-induced hydrothermal anomaly over the Earth's three Poles amplifies concurrent extremes in 2022. npj Climate and Atmospheric Science, 2023,7:8, DOI: 10.1038/s41612-023-00553-6."

** Visit Nature Portfolio's author and referees' website at <a href="http://www.nature.com/authors">www.nature.com/authors</a> for information about policies, services and author benefits**

Communications Earth & Environment is committed to improving transparency in authorship. As part of our efforts in this direction, we are now requesting that all authors identified as 'corresponding author' create and link their Open Researcher and Contributor Identifier (ORCID) with their account on the Manuscript Tracking System prior to acceptance. ORCID helps the scientific community achieve unambiguous attribution of all scholarly contributions. You can create and link your ORCID from the home page of the Manuscript Tracking System by clicking on 'Modify my Springer Nature account' and following the instructions in the link below. Please also inform all co-authors that they can add their ORCIDs to their accounts and that they must do so prior to acceptance.
https://www.springernature.com/gp/researchers/orcid/orcid-for-nature-research

For more information please visit http://www.springernature.com/orcid

If you experience problems in linking your ORCID, please contact the <a href="http://platformsupport.nature.com/">Platform Support Helpdesk</a>.

Version 1:

Decision Letter:

** Please ensure you delete the link to your author home page in this e-mail if you wish to forward it to your coauthors **

Dear Dr González-Herrero,

Your manuscript titled "Impact attribution of the March 2022 Antarctic heatwave reveals amplification by cloud feedbacks and increased future meltwater" has now been seen by our reviewers, whose comments appear below. In light of their advice we are delighted to say that we are happy, in principle, to publish a suitably revised version in Communications Earth & Environment.

We therefore invite you to revise your paper one last time to address the remaining concerns of our reviewers. At the same time we ask that you edit your manuscript to comply with our format requirements and to maximise the accessibility and therefore the impact of your work.

EDITORIAL REQUESTS:

Please review our specific editorial comments and requests regarding your manuscript in the attached "Editorial Requests Table".

*****Please take care to match our formatting and policy requirements. We will check revised manuscript and return manuscripts that do not comply. Such requests will lead to delays. *****

Please outline your response to each request in the right hand column. Please upload the completed table with your

manuscript files as a Related Manuscript file.

If you have any questions or concerns about any of our requests, please do not hesitate to contact me.

SUBMISSION INFORMATION:

In order to accept your paper, we require the files listed at the end of the Editorial Requests Table; the list of required files is also available at https://www.nature.com/documents/commsj-file-checklist.pdf .

OPEN ACCESS:

Communications Earth & Environment is a fully open access journal. Articles are made freely accessible on publication. For further information about article processing charges, open access funding, and advice and support from Nature Portfolio, please visit https://www.nature.com/commsenv/open-access

At acceptance, you will be provided with instructions for completing the open access licence agreement on behalf of all authors. This grants us the necessary permissions to publish your paper. Additionally, you will be asked to declare that all required third party permissions have been obtained, and to provide billing information in order to pay the article-processing charge (APC).

Please use the following link to submit the above items:
Link Redacted
** This url links to your confidential home page and associated information about manuscripts you may have submitted or be reviewing for us. If you wish to forward this email to co-authors, please delete the link to your homepage first **

We hope to hear from you within two weeks; please let us know if you need more time.

Best regards,

Haoyu Jiang, PhD
Editorial Board Member
Communications Earth & Environment

ChenRui Diao, PhD
Associate Editor,
Communications Earth & Environment
Consulting Editor,
Communications Sustainability

********************
REVIEWERS' COMMENTS:

Reviewer #2 (Remarks to the Author):

I am satisfied with the changes made; the revised paper is significantly clearer and the updated analyses more convincing. I recommend publication.

Reviewer #3 (Remarks to the Author):

I Thank the authors for their detailed response and revisions to the manuscript. I recommend priority publication after minor revisions.
1) The caption for Figure 2 is excessively lengthy, with the phrase "Changes in the 17-19 March 2022 averaged" appearing repeatedly. It is recommended to provide a unified explanation before describing each subfigure, which would make the caption more concise.
2) In the description of the methodology, the abbreviation "firn air content (FAC)" is used. It is recommended to consistently use the abbreviation throughout the text and provide the full term upon its first occurrence.

** Visit Nature Portfolio's author and referees' website at <a href="http://www.nature.com/authors">www.nature.com/authors</a> for information about policies, services and author

benefits**

**Open Access** This Peer Review File is licensed under a Creative Commons Attribution 4.0 International License, which permits use, sharing, adaptation, distribution and reproduction in any medium or format, as long as you give appropriate credit to the original author(s) and the source, provide a link to the Creative Commons license, and indicate if changes were made.
In cases where reviewers are anonymous, credit should be given to 'Anonymous Referee' and the source.
The images or other third party material in this Peer Review File are included in the article’s Creative Commons license, unless indicated otherwise in a credit line to the material. If material is not included in the article’s Creative Commons license and your intended use is not permitted by statutory regulation or exceeds the permitted use, you will need to obtain permission directly from the copyright holder.
To view a copy of this license, visit https://creativecommons.org/licenses/by/4.0/

**Editor:**

**Dear Dr González-Herrero,**

**Your manuscript titled "Impacts of Antarctic heatwaves amplified by climate change through water vapor and cloud feedbacks" has now been seen by 2 reviewers, and we include their comments at the end of this message. They find your work of interest, but some important points are raised. We are interested in the possibility of publishing your study in Communications Earth & Environment, but would like to consider your responses to these concerns and assess a revised manuscript before we make a final decision on publication.**

Thank you very much for your and reviewer's feedback. Following reviewer's comments and guidance we made major changes in our manuscript. We believe that now the manuscript provides a more consistent and compelling storyline that will be of interest of the readers of Communications Earth & Environment. We took the opportunity as well to change the title to one more apropiate "Impact attribution of March 2022 Antarctic heatwave reveals amplification by cloud feedbacks and increased future meltwater". To be able to show the changes made we submit a version of the manuscript with tracking changes and we answer below each reviewers' queries. Our answers to the reviewers are shown in blue while the direct reproduction of the manuscript text is shown in orange. We hope that the new manuscript satisfies now the editors and reviewer's and we are open to further reviews.

Best regards,

Sergi on behalf of all the coauthors.

**Reviewer #2 (Remarks to the Author):**

**Review of "Impacts of Antarctic heatwaves amplified by climate change through water vapor and cloud feedbacks" by González-Herrero et al., 2025**

**This paper investigates the influence of climate change on an Antarctic heatwave event that occurred in 2022, using a high-resolution coupled snow-atmosphere model. They find that water vapor radiative effects and cloud feedbacks amplify the near-surface warming in the present climate relative to the preindustrial, with implications for the regional surface mass balance. This is an interesting paper that explores feedbacks and processes that may be underrepresented in coarser GCMs. However, I have concerns about some of the analyses, particularly the heat and moisture budgets. I also think some of the figures could be improved and condensed, and that the authors should better address the differences in lower-level structure between their simulations and the observations (Extended Figure 1). For these reason I suggest this paper undergo major revisions.**

We thank the reviewer for her/his comprehensive review. We reviewed the entire manuscript following all the comments' made. We now justify better the simulations and analyses, and provide quantitative results for the attribution. We believe that the current version is more coherent and show better the physical changes produced in the event due to climate change. Find below the point-by-point answer to each comment.

**Major comments (scientific):**

**#1. The budgets for temperature and moisture tendencies are performed at different levels – sigma level 25 for temperature (~1800m) and sigma level 2 for moisture (~50m). The authors state in the methods that the temperature tendency analysis is conducted at a higher level to "assess processes above the atmospheric boundary layer," while the moisture tendency is done near the surface to examine "near-surface advection of moisture." However, since atmospheric rivers transport both moisture and heat together in the lower levels of the troposphere, the difference in height between the analyses is unintuitive, and also makes it difficult to compare the magnitudes of the effects. Given that the event being studied is a near-surface level temperature anomaly, it would make more sense to do budgets for both variables closer to the surface for consistency.**

Thanks for the comment. We agree that the previous presentation may have been confusing, and we have, therefore, redone the entire budget analysis following the reviewer's suggestion. To avoid clutter in the plots caused by misaligned fluxes when using a single level (see minor comment regarding this question), we calculated the budgets over a bulk layer between ~20 and ~80m. The selected heights were selected to remain below 100m, so we avoid interference with the layer where cloud formation and associated cooling occur, as shown in the vertical profiles over Concordia (Fig. 2b).

To further increase the robustness of our results, we selected a physically meaningful region where the amplification was non-linear (Fig. 1a) and calculated the average and dispersion over the region (see answer in major comment #4). We presented these results in Table 1 and Fig. 3. As a consequence of this revised analysis, the associated interpretation and discussion have also been substantially modified, with the paragraph below reflecting the most important changes:

We examined the drivers that lead to the amplification of the temperature around Dome C by comparing the current and the preindustrial simulations. During the diverging phase of the event (18 March) the temperature tendency in the lower atmosphere (~20-80 m above the dome) was 0.22 ± 0.02 °C h-1 higher in the current simulation with respect to the preindustrial one. Analysis of individual contributors reveals enhanced contributions from horizontal temperature advection (0.68 ± 0.07 °C h-1) and diabatic heating (0.72 ± 0.17 °C h-1), which were partially offset by adiabatic cooling (-1.18 ± 0.19 °C h-1) (Table 1, Extended Fig. 2a,b). Low level moisture increased by 0.6 g kg-1 in the current simulation with respect to the preindustrial one, primarily driven by horizontal advection (0.09 ± 0.01 g kg-1 h-1), being partially offset by reduced vertical advection (-0.06 ± 0.02 g kg-1 h-1) from higher levels (Table 1, Extended Fig. 2c,d and Supplementary Fig. 1.2). Overall, these results suggest that

temperature amplification over Dome C arises from approximately equal contributions of horizontal advection and diabatic effects, concurrent with a large horizontal moist advection.

**#2. In both the temperature tendency budget and the surface energy flux budget, the influence of horizontal/vertical sensible heat flux is not discussed in the text, despite being quite high in magnitude (Extended Figure 2, Supplementary Figure 2.1). ARs transport large amounts of sensible heat and cause anomalous downwards sensible heat flux at the surface, which is part of why they can cause such extreme surface temperature anomalies (e.g., Scholz & Lora, 2024). There are also obvious links between the changes in horizontal sensible heat transport and the surface flux changes that are not discussed. These results require further discussion in the text.**

Thanks for this comment and for pointing us to the relevant reference. Curiously, in our simulations, the sensible heat (SH) contribution during the event (Extended Fig. 2d) shows relatively weak values, specially in the windward side of the AR. While AR mobilize large amount of near-surface sensible heat, which cause anomalous downward sensible heat fluxes, we believe that in Antarctica this effect is partially offset by the persistence of a shallow, stably stratified cool katabatic winds. This is indeed consistent with Scholz & Lora (2024) article, which show lack of increased SH fluxes over Antarctica (see their Fig3i).

In any case, the primary focus in this study is not the thermodynamic perturbations induced by the AR itself, which are already well documented, but the changes that the AR is experimenting under global warming. Therefore, we provide SH fluxes mainly for reference. In the revised article with quantitative results (see answer to major comment #4) we can now show that the warming produce a slight decrease for the surface heat flux over the Dome C region when averaged spatially. However spatial patterns (Extended Fig 2p) show a decrease in the windward side of the dome and an increase in the leeside.

With all the revision made in response to the reviewer's comments, our manuscript is now more robust and provide more quantitative values and clearer physical links. To preserve the logical flow of the paper, the discussion of the SH fluxes has been kept concise. The more direct changes in the text regarding the SH reads now like this:

Despite ARs are known to enhance heat fluxes31, the global warming response leads to a redistribution of this flux, with reduced sensible heat uptake on the windward side and enhanced release on the leeward side (Extended Fig. 3d). The result is an average positive effect of 1.4 ± 1.1 Wm-2 over the Dome C region (Extended Fig. 3p).

**#3. As shown in Extended Figure 1, the simulations are warmer and have a different lower-level structure than observations, particularly the height of the inversion.**

**This seems a substantial discrepancy to me, especially when considering the influence of this inversion height on the cloud processes which are invoked in the paper. Further justification of these differences is needed.**

We appreciate the importance of this point and agree that the representation of the near-surface inversion deserves careful consideration. CRYOWRF exhibits a warm bias and a higher simulated inversion layer compared to observations at Concordia. Such warm bias is well documented for WRF and other models over the Antarctic plateau (e.g. Tastula & Vihma, 2011; Kurita et al. 2025; Gossart et al. 2019) and it is in fact larger in the CESM1-CAM5 simulations used for comparison (see Fig. 5c in Blanchard -Wrigglesworth et al. 2023, which report a minimum temperature in the model of -57 ºC on 12-March compared with our simulation with -59ºC (observations were -63ºC).

Importantly, this bias is largely time-invariant and present both before and after the heatwave. As a result, it does not drive the simulated amplification during the event itself. Indeed, the modeled temperature increase (~47 °C) remains smaller than observed (~51 °C), indicating that the bias acts against, rather than in favor of, the magnitude of the simulated amplification. At the same time, the warming capture by CRYOWRF is substantially larger than that reported in the CESM1-CAM5 storylines (~33 °C), suggesting that the higher-resolution, non-hydrostatic framework allows to capture key processes more realistically.

The model accurately reproduces the timing, duration, and moisture advection associated with the atmospheric river, and also reproduces the observed vertical moistening during the event. A further conceptual analysis lead us to realize that the impact of this elevated inversion is not unidirectional: the elevated inversion can enhance turbulent exchange and increase near-surface humidity, enhancing cloud formation, but at the same time spread the clouds over a thicker layer, reducing the LW radiation of the same clouds. We acknowledge this in the revised text.

Although we cannot fully quantify the impact of the inversion-height bias on cloud–radiation interactions, the arguments above indicate that the feedback mechanisms proposed in this study are physically plausible and likely operate in reality, even if their precise magnitude remains uncertain.

We modified the text accordingly in different sections to adapt it and explain better how our model responds to these limitations. The main changes are in the Model evaluation section, which reads now as:

Model evaluation

The 'current' run has been evaluated against surface temperature, as well as temperature and moisture profiles recorded by the Agenzia nazionale per le nuove tecnologie, l'energia e lo sviluppo economico sostenibile (ENEA) at Concordia station70,71, where the maximum temperature amplification was observed. The extensive measurements available at this site provide a detailed assessment of the model's performance. Our model exhibits a warm bias during the stable initial period (10-15 March) and simulates the near-surface temperature inversion at a higher elevation

than observed. Such discrepancies are common in models over the Antarctic plateau and primarily reflect limitations in representing extremely shallow inversions. However, both the duration of the heatwave and the maximum temperature in the current simulation align well with observations [Extended Fig. 1c]. This bias remains consistent throughout most of the temperature profile indicating internal consistency in the model [Extended Fig. 1a] and is present after the heatwave suggesting that does not control the event amplification analyzed here. A comparison of the water vapor mixing ratio before and during the heatwave event shows that the model accurately simulates the water vapor advection driven by the AR [Extended Fig. 1b]. The only notable discrepancy that may affect our results arises from the height of the near-surface temperature inversion, which is simulated higher than observed. This elevated inversion may influence the detailed coupling between clouds, moisture and the surface. However, the direction of this effect is not straightforward. While weaker surface inversion can enhance turbulent exchange and increase near-surface humidity and cloud formation near the ground, the same enhanced mixing can also spread the cloud layer over a deeper vertical extent, thereby reducing their radiative efficiency at the surface. Nevertheless, the model successfully captures the overall event dynamics and the primary drivers of the AR-induced warming, providing us with confidence on the results presented here.

References:

Tastula, E. M., & Vihma, T. (2011). WRF model experiments on the Antarctic atmosphere in winter. Monthly weather review, 139(4), 1279-1291.

Kurita, Naoyuki, et al. "Summer warming in the East Antarctic interior triggered by southern Indian Ocean warming." Nature Communications 16.1 (2025): 6764.

Gossart, A., Helsen, S., Lenaerts, J. T. M., Broucke, S. V., Van Lipzig, N. P. M., & Souverijns, N. (2019). An evaluation of surface climatology in state-of-the-art reanalyses over the Antarctic Ice Sheet. Journal of Climate, 32(20), 6899-6915.

Blanchard-Wrigglesworth, Edward, et al. "The largest ever recorded heatwave—Characteristics and attribution of the Antarctic heatwave of March 2022." Geophysical Research Letters 50.17 (2023): e2023GL104910.

**#4. Some assessment or measure of the statistical significance of results is needed. Particularly when the changes between experiments are only minimal, it would be useful to know how significant (or not) those changes are.**

We agree that some assessment of robustness is important. However, the PGW framework is deterministic by design and does not allow for a formal estimation of uncertainty through ensemble spread. Instead, our goal is to assess the physical consistency of the response to climate change for a specific event.

To address this, we quantified the robustness of the simulated changes by evaluating the spatial distributions over a physically meaningful region where the amplification was non-linear. Thus, we are able to provide a quantitative measure of changes on atmospheric variables and drivers without relying anymore on individual grid points, and also assess if the simulated changes exceed the internal spatial variability.

These values are represented in the new Table 1, as well through the text. Furthermore we specified this assessment in the methods section as:

Because the PGW approach relies on small number of deterministic simulations, it does not permit formal uncertainty quantification based on ensemble statistics. Instead, we assess the robustness of the simulated response by analyzing the distribution of the climate change response over the Dome C region (Fig. 1). This provides a quantitative measure to assess whether simulated changes exceed the internal spatial variability (see Table 1). Extended Table 1 and Supplementary Fig. 3.1 show the mean climate deltas over the whole model domain.

**Major comments (figures/formatting):**
**#1. Maps should have latitude/longitude to orient the viewer. It may also be helpful to add topographic contours to Figure 1, or to have a label or shading that shows Dome C for reference.**

We added topographic contours to Figure 1 for reference. Adding latitude/longitude contours to Figure 1a includes too much information and makes it more difficult the interpretation. Therefore, we included an inset with the map of the full Antarctica and the area depicted for reference. While the dome C has no clear boundaries, we selected a region of maximum amplification called Dome C region to calculate quantitatively the results (See answer in major comment #4).

**#2. There are additional figures presented as both "Extended Figures" and "Supplemental Figures." It is not clear to me what criteria was used to decide which figures go in which category, as often the supplemental figures are cited more in the text that the extended data figures. I would suggest putting all the figures most relevant to the main text in the extended data if possible. In addition, some supplemental/extended figures that are not referenced or relevant to the main text could be removed or condensed to ease interpretation.**

We have rearranged the figures in the manuscript. We have done 3 groups:

1. The main figures are maintained concise. They contain the direct attribution (Figs 1 and 5), the most relevant physical links (Figs 2 and 3) and the most relevant impacts on the firn (Fig 4), since they are one of the main novelties in the paper. We included now two tables with quantified results.

2. For the extended figures we included specially all those relevant with the PGW experiment, either showing secondary physical mechanisms (Extended Figs 2, 3 and 4) and secondary impacts (Extended Figs 4 and 5). We also include the comparison with observations which we think is still relevant.

3. In supplementary figures we show now only supporting figures which does not provide information on the results from the PGW, but on the conditions of the event and data used to initialize the PGW experiments.

We think that now the figure aggrupation follow a logic criteria for the text interpretation.

**Line comments:**

**L42. 25% of the event's magnitude – how much of an amplification is this, compared to the value you find?**

Here, the 25% indicates that 0.4 represents this percentage of the total 6-day and 2-day event anomaly in the analog events in both studies. To avoid confusions we rephrased it as:

Both studies quantified the contribution of climate change to be approximately 0.4 °C during the heatwave period, representing around 25% of the historical magnitude of comparable analog events.

**L47. Similarly, how much amplification does this 2C figure represent?**

Here we include as well that these 2ºC are:

"equivalent to about 6 % of the total magnitude of the simulated 30 °C anomaly event."

**L49. A brief description of what the analog approach vs. the storyline approach are would be helpful here, before the limitations of both methods are discussed.**

Thanks for the suggestion. We rephrased in the previous paragraph as:

It was assessed using an analog approach[9,10], comparing a set of circulation-constrained analogs in the historical record.

and:

For the March 2022 event, a storyline approach was used. Instead of relying on event probabilities, the authors nudged a global climate model circulation toward observations under different forcing scenarios[6] and examined their differences.

**L66. 'create detailed storylines' – please clarify what this means in the context of the model**

We rephrased as:

This allows us to construct physically consistent evolutions of the event, i.e. storylines,

**L81. Label Dome C on map.**

Dome C is a very well known structure in East Antarctica but it has no clear boundary definitions. Is mostly used to refer the region where the top heights altitude of the dome

are located but without specifying limits. Here we use it in this way since are exactly where the maximum amplification is located. In any case, in the new version of the manuscript we define it as a boundary depicted in Fig 1a that is used afterwards in different parts of the manuscript to quantitatively describe the amplification.

**L95-97. This sentence is unclear, what is meant by "no amplification under current climate conditions in altitude?"**

This part of the text has been removed due to the new analyses suggested in major reviews.

**L98. "No dominant driver above the boundary layer" but there is substantial horizontal sensible heat advection, though this seems to be somewhat balanced by diabatic cooling (further explanation of this would be helpful too). I do not think we can conclude from these figures that there is no higher-level influence on the warm surface temperatures, and it certainly does not exclude the role of sensible heat flux either.**

All this paragraph has been now replaced by a new argumentation following the new analyses done

**L110-112. This argument is not very clear, and the feature mentioned is not obvious in the plots referenced.**

We decided to delete this argument to not distract from the main argumentation.

**L119. 'Removing energy… on the windward side:' this is not exactly accurate, as the figure shows the difference between the preindustrial and current, so the negative values just represent 'less downward' sensible heat flux. Also, this needs a reference to the appropriate figure number.**

Thanks for the suggestion. We corrected the text and referenced the proper figure. Now it reads:

Despite ARs are known to enhance heat fluxes31, the global warming response leads to a redistribution of this flux, with reduced sensible heat uptake on the windward side and enhanced release on the leeward side (Extended Fig. 3d). The result is an average positive effect of 1.4 ± 1.1 Wm-2 over the Dome C region (Extended Fig. 3p).

**L126. This is the only time that Figure 3 appears to be cited, and only 3 components of it are mentioned.**

Following a request of the reviewer 2, we further enhanced the physical mechanisms that lead to more liquid water over the Dome C surrounding this figure and by including new ones. Figure 3 now takes even more relevance. The new paragraph now reads as:

A relevant question is why liquid droplets are present over the dome but not along the ascent path (Fig. 3f,h), where only ice-phase hydrometeors are found (Fig. 3j,l). Cloud particles associated with the atmospheric river are initially formed primarily as liquid over lower latitudes and gradually transition to mixed-phase near the Antarctic coast (Fig. 3f,h,j,l). As the air penetrates inland and cools, the liquid phase is largely depleted and converted into ice through freezing and deposition processes. During the ascent over the Antarctic Plateau, the continued large-scale lifting further cools the air mass, progressively reducing its moisture-holding capacity (Fig. 3b,d). Over the dome, weak winds and moisture convergence promote supersaturation with respect to both liquid water and ice, favoring the in-situ formation of supercooled liquid droplets from advected water vapor in a low ice nucleation particle environment. In the simulations, this behavior is influenced by the temperature-dependent ice nucleation parameterizations of the Morrison microphysics scheme. Although this may lead to quantitative uncertainties in phase partitioning, the resulting dominance of supercooled liquid water is physically plausible and consistent with observations over the Antarctic Plateau32. Observational studies indicate that extremely low ice nucleation particle concentrations suppress efficient heterogeneous ice nucleation32,33, allowing liquid droplets to persist and relaying primarily on homogeneous freezing at very low temperatures33,34. Continuous vapor supply maintains these droplets, while ice growth and precipitation remain inefficient, producing a localized maximum in liquid water path over Dome C. Our simulations suggest that current warmer conditions enhance this effect with respect to the past (Fig. 3c,g,k).

**L127-128. Are there relevant topographic reasons why this is occurring?**

Following a request of the reviewer 2, we further enhanced the physical mechanisms that lead to more liquid water over the Dome C by including new figures and developing the argumentation in the previous paragraph. The reasons are more about the cloud physics than topographical, although topography plays a small role providing lifting to the air.

**L129. Clarify here that you are referring to the exact proportion of LWP vs IWP, etc., otherwise it seems to contradict the budget analysis. Additionally, given the changes in LWP/IWP/etc., isn't there a way to estimate the estimate the longwave contribution of these different factors?**

We clarified in the text that we are referring to the partition between water vapor, ice clouds, and liquid clouds. CRYOWRF outputs only contain the radiative aggregate of all the components in the atmosphere. The exact radiative effect of each component does not only depend on the quantity but on the altitude, they are and other factors such as the sun angle. Therefore, without using a radiation transfer model is not possible to quantify the exact partition in each component. We now acknowledge this in the text which reads now as:

While the exact partition between water vapor, ice clouds, and liquid clouds cannot be quantified without dedicated radiative perturbation experiments, the spatial coincidence of the longwave radiation maximum with enhanced liquid water path over Dome C (Fig. 3f) indicates that supercooled liquid clouds act as a key local amplifier of the near-surface warming.

**L131. 13-43 g m-2 of supercooled water?**

Corrected, thanks.

**L135. This doesn't exactly confirm it, but provides some strong evidence suggesting that it is a plausible cause.**

We agree that we cannot directly demonstrate this. We softened the claim saying:

These findings provide strong support for the hypothesis that supercooled liquid droplets may be the main driver in the non-linear temperature amplification.

**L151. "redistribution between the two experiments" – unclear, please clarify.**

Thanks for noticing. We agree that is unclear. We specified that we talk about snow redistribution.

likely resulting in minor differences in snow redistribution between the two experiments

**L158. Why does the density remain lower in the preindustrial simulation, and what implications does this have?**

Lower snow loading leads to lower compaction. These changes are still small and can have some small implications in firn physical properties mentioned, but still is an interesting exercise to see how firn responds to climate change. To accommodate this explanation we rephrased as:

After subsequent snow compaction, snow density increases above the initial conditions in the current simulation but remains lower in the preindustrial simulation [Extended Fig. 5b]. The lower density in the preindustrial case arises from the absence of the modest precipitation increase seen in the current climate, which limits snow loading and therefore reduces post-depositional compaction. While resulting density differences are relatively small, they modify the snow thermal gradient and firn physical properties, influencing firn metamorphism and the exchange of water isotopes with the atmosphere40.

**L181. This argument is unclear and needs further explanation, please clarify. In addition, wouldn't a warmer world lead to a change in the 'physical limit' for supercooled water effects over the dome?**

We acknowledge that the word physical limit may be confusing. We were referring to a limitation in the non-linear phase of the amplification and the return to a consistent-to-background linear amplification. To clarify our explanation we rephrased the full paragraph as:

In these simulations, changes are not only driven by the increase of supercooled liquid water but by increases in all the vertically integrated water components, including water vapor and ice cloud droplets (Extended Fig. 7), due to a stronger horizontal moisture advection towards the continent and higher extension of ice clouds (Extended Fig. 8) probably driven by a warmer atmosphere and reduced

sea ice extent. Importantly, the spatial pattern of non-linear temperature amplification evolves with the background climate state. In the transition from preindustrial to current conditions, amplification is strongest over the cold interior, including Dome C. In contrast, in future simulations relative to the current climate, the non-linear amplification progressively weakens over the plateau and shifts toward lower-elevation regions, first between the interior and the coast (SSP2-4.5) and ultimately becoming confined to coastal areas under stronger warming (SSP5-8.5). The return to the linear background warming signal suggest a saturation of cloud-radiative feedbacks and the progression towards the coast suggest that cloud–radiative feedbacks operate most efficiently within a specific temperature and moisture regime, which migrates spatially as the Antarctic climate warms (Fig. 5a,b).

**L185. More details about why firn air content is a relevant or important metric would be helpful here.**

We started the paragraph giving a short explanation on the importance of the firn. But instead here we did it in section 2.2 where firn air content explanations first appear.

These structural changes in the firn layer are accompanied by a broader firn response, including changes in firn air content, which provides an integrated measure of pore space and controls the capacity of the firn to store meltwater and buffer surface meltwater inputs.

**L199. Why are liquid-bearing clouds less prevalent than in the Arctic?**

We modified as:

Compared to the Arctic, liquid-bearing clouds are less prevalent and persistent in Antarctica due to colder temperatures and less ice-nucleating particle concentration40

**L207-210. Yes, the peak temperature is closer, but the CRYOWRF simulations are also several degrees hotter in general, which makes this statement less convincing. See major comment #3.**

We justify this fact in the answer to the major comment #3

**L213. The impact of the hydrostatic approximation in GCMs is mentioned several times in the intro and conclusion. However, the exact influence of using a non-hydrostatic scheme in this model is not explicitly stated; how would the results be different if hydrostasy was assumed? The vertical motion of sensible heat seemed to play a minimal role of the budget, for example.**

Thanks for this clarification. After the new analyses done, we can see that the vertical motion plays an important role both in temperature and moisture tendency in the lower layer. We clarified in the text accordingly:

In typical Global Climate Models (GCMs) like NCAR CESM1-CAM5, coarse grid resolution and the hydrostatic approximation strongly simplifies the representation of vertical motions, requiring cloud microphysics and turbulent processes to be parameterized over large grid volumes. In our

simulations, vertical moisture advection increase is of comparable magnitude to horizontal advection during the event (Table 1), enabling enhanced formation and persistence of supercooled liquid water clouds within shallow inversion layers—processes that would be strongly damped in a hydrostatic model. These processes, which are among the most poorly constrained aspects of the models, lead to suppression of key processes that high-resolution non-hydrostatic models can explicitly resolve51. In addition, the lower vertical resolution may suppress key layers and atmospheric inversions.

**L217. The link between ARs and supercooled liquid water clouds is not clear.**

We explicated the physical link between atmospheric rivers, low-level inversions and persistence of supercooled liquid water clouds more clearly by explicitly stating the role of moisture transport and cloud formation. New text states:

These limitations are especially relevant in very strong advective events as seen in ARs, which transport large amounts of moisture into Antarctica and promote cloud formation within strong low-level inversions52, favoring the persistence of supercooled liquid water clouds that enhance longwave downward radiation53.

**L244-246. Run on sentence, please rephrase for clarity.**

We splitted the sentence in two for clarity:

We therefore present these results as a process-based exploration of plausible responses. They should be interpreted as complementary to large-scale GCM ensembles, rather than probabilistic estimates bridging weather-scale processes with climate-scale projections.

**L247. This argument seems contradictory - in L225, you state that CMIP6 models overestimate the occurrence of these clouds, but here you state that the amplification due to these clouds is largely absent from current GCMs? Please clarify.**

We agree with the reviewer that this argument may look contradictory. However, the increased presence of supercooled liquid clouds in CMIP6 models does not guarantee a non-linear radiative amplification, since the vertical structure, persistence and coupling with shallow inversions during extreme events also controls the changes in downward longwave radiation. We clarified this in the discussion on how GCM responds to liquid water. Now we say:

For example, a recent study57 compared supercooled liquid-containing clouds in CMIP6 models with satellite observations and found that CMIP6 models consistently overestimate their occurrence leading to light snowfall being produced too frequently. This excessive snowfall maintains an artificially bright snow surface over ice sheets enhancing albedo and causing GCM to underestimate surface melt. Furthermore, liquid water cloud presence does not necessarily translate into a realistic radiative response during extreme events. The non-linear surface warming identified here arises from the tight coupling between moisture advection, sharp low-level inversions, and the vertical confinement of supercooled liquid water clouds, which strongly enhances downward longwave radiation over short

time scales. In contrast, parameterized cloud schemes in GCMs tend to distribute liquid water more diffusely in the vertical direction and produce more persistent cloud fields, weakening their interaction with near-surface inversions and smoothing the radiative response58,59. As a result, even when supercooled liquid clouds are present in nudged simulations with GCMs, their event-scale radiative amplification may be underestimated. These biases arise from poor representation of cloud microphysics, further highlighting the need for model improvements in cloud microphysics and sub-grid processes.

**L305-307. Given that this paper focuses on water vapor and cloud feedbacks, this seems to be a critical difference. See major comment #3.**

We rewrote the text accordingly. See answer in major comment #3

**L353. See major comment #1 about the temperature and moisture budgets.**

We rewrote the text to reflect the changes explained in major comment #1

**L358. In addition to the horizontal and vertical advection, local convergence of both sensible heat and moisture are important in the lower-level dynamics of ARs, playing a role in warming the surface.**

Thanks for this suggestion, which is very interesting. We quantified both local moisture convergence and sensible heat convergence in the lower troposphere. Moisture convergence is briefly discussed and shown in Fig. 2d, as it directly contributes to the thermodynamic amplification emphasized in this study. Sensible heat convergence exhibits a similar spatial pattern, largely driven by low-level wind convergence/divergence associated with the same flow as moisture converence. However, its contribution is secondary relative to radiative and moisture-related processes we discuss here and we therefore excluded it from the analysis.

**L365. The residual "Diab" term here could include a large variety of effects, which may not be appropriate to group together. I would consider this breakdown more carefully. Adiabatic and diabatic effects can also be estimated separately (e.g. Röthlisberger & Papritz, 2023).**

We acknowledge that this Diab term include other variety terms as already explained in the text. We agree that the methodology of Röthlisberger & Papritz (2023) provide a more detailed decomposition but this would require a lagrangian approach that we do not use in this study. This approach would be computationally intensive for the purpose of our study. We therefore retain the current approach, which is consistent with similar studies (e.g. Jimenez-Estebe & Domeisen, 2022; Hori et al., 2024; Sousa et al. 2020; Chen et al. 2024) and sufficient to interpret the relative contributions of advection and diabatic processes, while acknowledging in the text that interpretation has to be taken carefully.

Notice that the last term also includes other non-accounted terms like the diffusive fluxes and computational errors, which can be further decomposed using Lagrangian methods (reference

Röthlisberger). For our purposes we employ a conventional Eulerian decomposition supposing that these non-accounted terms are of an order of magnitude lower; however, interpretation of residual values have to be taken with caution. . This equation was evaluated at low levels, between the sigma level 2 and 5 (around 26 - 82 m over Concordia station at the top of Dome C).

Jiménez-Esteve, B., & Domeisen, D. I. (2022). The role of atmospheric dynamics and large-scale topography in driving heatwaves. Quarterly Journal of the Royal Meteorological Society, 148(746), 2344-2367.

Hori, M., Yoshimori, M., & Ukita, J. (2024, December). Changing role of horizontal moisture advection in the lower troposphere under Arctic amplification using a large-scale ensemble model dataset. In AGU Fall Meeting Abstracts (Vol. 2024, No. 1962, pp. A33B-1962).

Sousa, P. M., Barriopedro, D., García-Herrera, R., Ordóñez, C., Soares, P. M., & Trigo, R. M. (2020). Distinct influences of large-scale circulation and regional feedbacks in two exceptional 2019 European heatwaves. Communications Earth & Environment, 1(1), 48.

Chen, W., Wei, W., & Yang, S. (2024). Moisture dynamic processes of the westward propagation of quasi-biweekly oscillation in Asian tropical summer monsoon rainfall. Atmospheric Research, 310, 107639.

**L373. These components at different vertical levels are not shown or referenced.**

This sentence has been removed after changes in the analysis methodology

**Extended Figure 4. In both this figure and Figure 6, the changes in FWC and pILT are very small. Further information on why these spatially-limited changes are relevant would help make their importance more clear.**

Thanks a lot for this comment. We agree that maybe showing only maps do not show the real magnitude of the changes, we therefore proceed to quantify the magnitude of the changes in Table 2. The table show that the changes are much important as the figure may suggest since changes in FWC have already increased 2 times and can futher increase 2.4 and 5.4 times by the end of the century with respect future conditions under SSP2-4.5 and SSP5-8.5 scenarios, respectively. We modified different sections as:

While the event itself drives substantial changes in FAC, our attribution analysis indicates that climate change has only a minor effect on firn air [Fig. 4a], but still producing a two-fold increase in firn liquid waternear Wilkes Land from 0.008 Gt in the preindustrial conditions to 0.014 Gt in the current conditions [Table 2; Fig. 4c]. This small addition of liquid water is sufficient to slightly increase the potential ice lens thickness under current climate conditions relative to preindustrial conditions [Fig. 4f].

Similarly, firn liquid water content rises by 4 cm in the SSP2-4.5 scenario and by up to 10 cm in the SSP5-8.5 scenario, with the affected areas expanding accordingly in warmer scenarios (Fig. 6e,h). The associated meltwater production increases dramatically and non-linearly. While the current

simulation produces only 0.012 Gt, future projections show increases by factors of 2.4 and 5.6 under SSP2-4.5 and SSP5-8.5, respectively (Table 2). This single event alone would generate up to 0.079 Gt of meltwater by the end of the century under the SSP5-8.5 scenario. The excess liquid water further enhances potential ice-lens formation within the firn, increasing ice-lens volume by up to 0.01 $km^3$ (Table 2; Fig. 6f,i). These values represent lower bounds, as a fraction of the meltwater has not yet fully percolated by the end of the simulation and would continue to refreeze with additional settling time. The resulting reduction in firn permeability favors meltwater ponding and promotes hydrofracturing. These processes are known to weaken ice-shelf stability[19], highlighting the potential consequences that future AR-driven heatwaves may exert along Antarctic coasts.

We therefore included the main results explicitly in the abstract as well.

**Supplement: The figure showing changes in temperature tendency from preindustrial to current (Supplementary Figure 2.1) appears to be very noisy – why is this?**

This is a typical behavior in non-hydrostatic models at relatively high resolution. When subtracting two fields from current and preindustrial, small changes in winds, local instabilities and amplified vertical motions can misalign fluxes. These changes can be indeed physically meaningful and reveal fine scale variability. However, due to the challenge that supposed in interpretation, in the new version of the manuscript we evaluated the tendency equations to a bulk layer at low levels following the answer to major comment #1, which reduce this noisy pattern.

**References:**
**Scholz SR, Lora JM. Atmospheric rivers cause warm winters and extreme heat events. Nature. 2024 Dec;636(8043):640–6.**
**Röthlisberger M, Papritz L. Quantifying the physical processes leading to atmospheric hot extremes at a global scale. Nat Geosci. 2023 Mar;16(3):210–6.**

**Reviewer #3 (Remarks to the Author):**

**The manuscript " Impacts of Antarctic heatwaves amplified by climate change through water vapor and cloud feedbacks "  investigates the climate change contribution to the March 2022 East Antarctic heatwave using pseudo-global warming experiments with the snow-atmospheric coupled model CRYOWRF. It is significant due to land ice loss under global warming, however, the manuscript is should make major revisions as below.**

Thank you for the insights raised during the review. We specially acknowledge that the question 4 was very important and helped us to provide new analyses to better physically connect the findings from our PGW simulations. We believe that now the manuscript provides a much better storyline and physical insights. Find below point-by-point answers to every query.

**1) Figure 2. b-c is vertical profile in lower troposphere atmosphere? How to understand column axis unit?**

Panels b and c in Figure 2 shows the vertical profiles in the lower atmosphere in geometric coordinates [units in meters above ground level (m. a.g.l.l)]. It goes from 0 to 3000 m. a.g.l. which corresponds the lower troposphere over the Antarctic plateau. We clarified this in the caption:

Vertical profiles are function of height above ground level (m a.g.l.).

**2) How to define near-surface? Why not use 2m temperature?**

We indeed meaning 2m temperature in the plots. We agree that it can be confusing, specially because we also use this term for close to the surface inversions, which are not directly defined in terms of height. Therefore, we modified the manuscript and figures accordingly to clearly state 2m temperature instead of near-surface.

**3) In abstract:"linearly amplify near-surface warming by up to 25% relative to preindustrial conditions", how to get this 25%? In the second paragraphs in main, "representing around 25% of the events magnitude, Are there other other contributions such as adiabatic heating due to descending motion?**

Coincidentally, these two 25 % are different and unrelated values. In the abstract we refer to 25 % of temperature amplification during the event of March 2022 by all the mechanisms explained in the paper. In case of the introduction section, this 25 % represents the amplification calculated using the analog methodology for the events of Feb 2020 and March 2022 in the Antarctic peninsula, where current analogs are 25 % higher magnitude compared with past analogs.

**4) Fig 3. liquid water path over observation station seems an independent and is far from ocean, how to link with sources of liquid water?**

This is a very relevant question for the paper. In the previous version of the manuscript we did not specify the physical link between the sources of liquid water in the ocean and in the interior. LWP over the Dome is not directly connected to the oceanic region but forms in situ from the water vapor advected inland by the atmospheric river.

We discussed this in a new paragraph in section 2.1 saying:

A relevant question is why liquid droplets are present over the dome but not along the ascent path (Fig. 3f,h), where only ice-phase hydrometeors are found (Fig. 3j,l). Cloud particles associated with the atmospheric river are initially formed primarily as liquid over lower latitudes and gradually transition to mixed-phase near the Antarctic coast (Fig. 3f,h,j,l). As the air penetrates inland and cools, the liquid phase is largely depleted and converted into ice through freezing and deposition processes. During the ascent over the Antarctic Plateau, the continued large-scale lifting further cools the air mass,

progressively reducing its moisture-holding capacity (Fig. 3b,d). Over the dome, weak winds and moisture convergence promote supersaturation with respect to both liquid water and ice, favoring the in-situ formation of supercooled liquid droplets from advected water vapor in a low ice nucleation particle environment. In the simulations, this behavior is influenced by the temperature-dependent ice nucleation parameterizations of the Morrison microphysics scheme. Although this may lead to quantitative uncertainties in phase partitioning, the resulting dominance of supercooled liquid water is physically plausible and consistent with observations over the Antarctic Plateau32. Observational studies indicate that extremely low ice nucleation particle concentrations suppress efficient heterogeneous ice nucleation32,33, allowing liquid droplets to persist and relaying primarily on homogeneous freezing at very low temperatures33,34. Continuous vapor supply maintains these droplets, while ice growth and precipitation remain inefficient, producing a localized maximum in liquid water path over Dome C. Our simulations suggest that current warmer conditions enhance this effect with respect to the past (Fig. 3c,g,k).

**5) Some possible drivers should be discussed that warming further intensifies this amplification of near-surface warming.**

In the new version of the manuscript we quantitatively addressed the drivers that intensifies the amplification. The new Table 1 and Extended Fig. 3 and Fig. 3, with all the changes made in section 2.1 presents now a more complete picture of the amplification.

**6) Previous studies shows ice cloud contribute warm, however water vapor is also a kind of greenhouse gases, In Fig.3, you talk about ice water path and liquid water path, can you shows the contributions of them to near-surface temperature?**

We agree that water vapor is an important greenhouse gas and contributes to near-surface warming together with clouds. In the manuscript, water vapor changes are explicitly analyzed. The near-surface warming reflects the combined longwave greenhouse effect of vapour and clouds. While a quantitative separation of their individual radiative contributions would require dedicated radiative perturbation experiments, which are beyond the scope of this storyline framework, the spatial coincidence of the maximum downward longwave radiation anomaly with enhanced liquid water path over Dome C suggests that supercooled liquid clouds act as a key local amplifier of the warming. We have clarified this in the revised text as:

In our simulations, the combined effect of these changes lead to a substantial increase of downward longwave radiation of 10-40 W m-2 over Dome C, with much weaker increases away from Dome C. While the exact partition between water vapor, ice clouds, and liquid clouds cannot be quantified without dedicated radiative perturbation experiments, the spatial coincidence of the longwave radiation maximum with enhanced liquid water path over Dome C (Fig. 3f) indicates that supercooled liquid clouds act as a key local amplifier of the near-surface warming. This interpretation is consistent with Arctic observations and modelling studies showing that increases in liquid water path of 13 - 43 g m-2, can lead to an increase of downward longwave radiation by 40 W m-2 over the surface35. Recent observational studies at Dome C further demonstrate that cloud liquid water content increases logarithmically with temperature producing a similar radiative impact in downward longwave radiation36,37. These findings provide strong support for the hypothesis that supercooled liquid droplets may be the main driver in the non-linear temperature amplification.

**7) Figure 4 SMB seems the result of near-surface temperature or global warming forcing on surface energy and SMB, it is far from topic, I suggest to delete it.**

Thanks for the suggestion. We think that the figure is important since it quantifies for first time the changes in SMB due to global warming for an extreme event, and a big part of the article focuses on the impact attribution. This is especially important in Antarctica, where SMB drives great part of the ice sheet. Still, as the reviewer mention is not a fundamental part of the study. Therefore, we decided to downgrade the figure as Extended Figure but still maintain it.

**8) Fig.5, near surface temperature is so lower, how to define heatwave of positive temperature in 2022 ? Is it suitable?**

The term heatwave depends on the definition used and it can be defined in terms of statistical rarity, intensity, persistence, physical threshold exceedance, multivariate combinations of drivers or the magnitude of the resulting impacts. One of the most used definitions by the WMO is indeed in terms of percentile. For example, one of most widely definitions is "a period of at least three consecutive days when both the daily surface air temperature minima and maxima are higher than the highest 5% of values for the day in question during the 1991–2020 reference period" used in the European State of the Climate among other places. Using this definition, and others whatever part of the world is subject to heatwaves and the temperature threshold depends on the place where it occurs. In Antarctica, the term heatwave is widely used for either anomalous temperatures slightly above the melting point in coastal areas, and even in below-zero temperature as this case. Indeed it iwas already stated as the "largest ever recorded heatwave" by Blanchard-Wrigglesworth et al.

Blanchard-Wrigglesworth, E., Cox, T., Espinosa, Z. I., & Donohoe, A. (2023). The largest ever recorded heatwave—Characteristics and attribution of the Antarctic heatwave of March 2022. Geophysical Research Letters, 50(17), e2023GL104910.

**9) Antarctic warming effect is emphasized. Recommend a manuscript "Zhang J.*, H. Chen, X. Fang, Z. Yin, R. Hu. Warming-induced hydrothermal anomaly over the Earth's three Poles amplifies concurrent extremes in 2022. npj Climate and Atmospheric Science, 2023,7:8, DOI: 10.1038/s41612-023-00553-6."**

Thank you very much for the reference, it fits well to explain the consequences in the coastal regions. We included it at the end of the manuscript as:

This, in turn, triggers non-linear temperature amplification and intensifies AR-associated heatwaves near the coast, consistent, as well, with broader evidence that surface melt can further enhance warming during extreme events61.
